# Defining Recovery and Relapse in Bulimia Nervosa: A Systematic Review of the Literature

**DOI:** 10.1002/erv.70033

**Published:** 2025-09-24

**Authors:** Valentina Gardini, Francesca Pagli, Elena Tomba

**Affiliations:** ^1^ Department of Psychology University of Bologna Bologna Italy

## Abstract

**Objective:**

Despite advances in understanding bulimia nervosa (BN), standardized definitions of outcome stages remain lacking. This review aims to synthesise definitions of recovery and relapse in BN to improve its assessment and comparability across study outcomes.

**Methods:**

A systematic review was conducted using PRISMA guidelines. PubMed and PsycINFO were searched (October 2024) combining keywords ‘recovery’, ‘remission’ or ‘relapse’ with ‘bulimia nervosa’.

**Results:**

*N* = 76 studies were included. Recovery was most commonly defined using diagnostic (*n* = 30, 53%; For example, a Psychiatric Status Rating score ≤ 2 and absence of a DSM‐based diagnosis) or behavioural criteria (*n* = 28, 49%; typically binge eating and compensatory behaviours assessed with the Eating Disorder Examination interview/questionnaire; EDE/EDE‐Q). Fewer studies considered medical/physical (*n* = 17, 30%; That is, Body Mass Index ≥ 18.5) or psychological criteria (*n* = 10, 18%; For example, EDE/EDE‐Q global or all subscales score within 1SD of community norms). *N* = 11 (14%) addressed partial recovery as a period of symptomatic improvement with residual symptoms. Relapse was defined using behavioural criteria (*n* = 18, 25%; That is, re‐emergence of binge eating and compensatory behaviours) or meeting DSM‐based diagnostic criteria after remission (*n* = 10, 14%).

**Conclusions:**

Adopting multidimensional definitions of recovery and relapse, incorporating the most endorsed behavioural, diagnostic, medical/physical, and psychological criteria, may increase diagnostic accuracy, facilitate assessment and outcomes comparability.

## Highlights

- There is a lack of standardized definitions in the literature for recovery and relapse in bulimia nervosa, making it difficult to compare outcomes across studies and assess treatment outcomes accurately.
- Recovery from bulimia nervosa in the literature was most commonly defined by diagnostic criteria (a Psychiatric Status Rating score ≤ 2 or absence of a DSM‐based diagnosis) or the absence of core behavioural symptoms, such as binge eating and compensatory behaviours.
- Partial recovery from bulimia nervosa, which precedes full recovery, was most commonly defined as a period of symptomatic improvement with the persistence of residual symptoms.
- Relapse from bulimia nervosa was most commonly defined as the return of behavioural symptoms (i.e., binge eating and compensatory behaviours) or a meeting again the threshold for a DSM‐based diagnosis after a period of remission. This underscores the importance of targeted relapse prevention during this vulnerable period.

## 1 Introduction

Bulimia nervosa (BN) is a psychiatric disorder characterised by recurrent episodes of binge eating followed by compensatory behaviours to avoid weight gain (American Psychiatric Association 2022). This eating disorder (ED) is associated with compromised mental and physical well‐being, as well as impaired psychosocial functioning (Hay 2020). Lifetime prevalence rates of BN range from 0.3% to 4.6% (Hay 2020), with a high variability in course and outcomes among patients (Steinhausen and Weber 2009). Moreover, recent evidence from a large‐scale meta‐analysis (Solmi et al. 2024) has highlighted the heterogeneous and often suboptimal outcomes of EDs, including BN. Recovery occurs in fewer than half of the cases, and a significant proportion of individuals experience chronicity or relapse after initial remission, with only modest improvement over time.

To improve the effectiveness of interventions for BN, a deeper understanding of its course and progression is essential.

The staging model of psychiatric disorders is an approach that was developed in medicine, primarily oncology, and it describes a disease continuum with stages of increasing severity (McGorry and Mei 2021). The application of staging models to psychiatric disorders (Fava and Kellner 1993) enables the differentiation of severity, temporal progression, and clinical characteristics within the same disorder. In addition, the assessment of a patient's disease stage is crucial for making prognostic and treatment decisions, such as selecting interventions appropriate to the stage of illness. Staging also provides the basis for tailored interventions, prevention and disease trajectory prediction (McGorry and Mei 2021). Some authors (Cosci and Fava 2013; Treasure et al. 2015) applied the staging model also to the assessment of the longitudinal trajectory of EDs, which may help to better conceptualise the illness progression with regard to relapse, recovery and chronicity.

However, most staging models for EDs have primarily been applied to anorexia nervosa (AN) (Tomba et al. 2024), where the early phase of AN is followed by two to five stages characterised by a progression of psychological, behavioural, and physical symptoms. These stages can lead to recovery or to a persistent illness (Tomba et al. 2024).

For BN, only a few staging models have been developed. Specifically, Cosci and Fava (2013) proposed a four‐stage model for BN. The prodromal phase (stage 1) is marked by non‐specific symptoms, such as dietary restriction, low self‐esteem, or irritability. The second stage is the acute phase, characterised by a decrease in perceived control over eating and engagement in inappropriate compensatory behaviours. A residual phase (stage 3) may occur, in which some symptoms persist after the acute symptoms have remitted. Over time, the disorder may develop into an attenuated or persistent chronic form of BN (stage 4) if the residual symptoms worsen (Cosci and Fava 2013). Additionally, a five‐stage model of EDs, including BN, has also been proposed by Treasure et al. (2019). The first stage, High Risk, refers to the early phase when risk factors that increase vulnerability to the onset of an ED have been recognized. The second stage, Ultra‐High Risk, is characterised by individuals exhibiting ED prodromal symptoms such as binge eating, compensatory behaviour, fear of gaining weight, and hyper‐evaluation of weight and body shape (Stice et al. 2021). The Early Stage (stage 3) of BN is then defined, in which initial weight loss is observed and cycles of restrictive and compensatory behaviours may occur. These behaviours may progress to the Full Stage of the disease (stage 4), where individuals meet the full diagnostic criteria for BN. Ultimately, the illness may worsen over time and become more challenging to treat, with rigid habits causing impairments in the social, psychological, and medical domains. The final stage (stage 5) is chronicization, the Severe and Enduring stage of the disorder (Treasure et al. 2019).

Overall, there was not much agreement on how to define the stages in BN, which makes it difficult to improve knowledge of the illness's prognosis, management and treatment (Tomba et al. 2024; Treasure et al. 2015). This also led research on EDs outcomes (Ackard et al. 2014; Bardone‐Cone et al. 2018; Miskovic‐Wheatley et al. 2023) to underline that a wide range of definitions applying different criteria has been used to describe treatment outcomes and that various studies have used quite different conceptualizations of recovery, remission and relapse. Consequently, the evaluation of study results and the incorporation of data from other studies are more challenging due to the varied criteria applied, which result in significantly different recovery and relapse rates (Gorrell et al. 2020; Williams et al. 2012). Although proposals have been made to standardise definitions for AN (Khalsa et al. 2017), they have not been fully standardized for BN. Moreover, while most staging models for EDs or BN alone have emphasised progression towards chronicity (Treasure et al. 2015), integrating also phases of remission, recovery, and relapse in these models may help to more accurately reflect the fluctuating course of these illnesses. Clarifying how these phases are defined is essential for advancing staging models of BN and informing both clinical decision‐making and research.

Therefore, this paper aims to conduct a review to systematically examine how definitions of recovery and relapse in BN are conceptualised and operationalised in the literature, considering the importance of a consensual definition to facilitate the longitudinal assessment of this ED. Additionally, this systematic review aims to summarise BN recovery and relapse rates reported in the articles.

## 2 Materials and Methods

### 2.1 Search Strategy

This systematic literature review was conducted following PRISMA guidelines (Moher et al. 2009) on two databases (i.e., PubMed and PsychINFO). The keywords (relapse OR remission OR recovery) AND (bulimia nervosa) were used to identify articles providing definitions of recovery and relapse. The keyword ‘remission’ was included in order to ensure comprehensive coverage of studies as articles about remission were believed to also include definitions of relapse and recovery. However, only studies that provided an explicit operational definition of recovery and/or relapse were included in the systematic review. Studies that reported definitions of remission alone, without a corresponding recovery definition, were excluded. In cases where both terms appeared, only definitions specifically labelled as recovery were extracted and analysed.

The search was limited to articles written in English and published in peer‐reviewed journals. Duplicate articles were removed before titles and abstracts were screened by two authors (*V.G. and F.P.*). Articles that did not meet eligibility criteria or that were not relevant to the research topic were excluded. The same two authors then independently assessed the full texts of relevant studies for the review. In case of disagreement, the full texts were revised and discussed by a third author (*E.T.*) until consensus was reached. The review protocol was registered on the ‘Prospero International Prospective Register of Systematic Reviews’ (PROSPERO ID: CRD42024595941). The review was conducted in October 2024.

**FIGURE 1:**
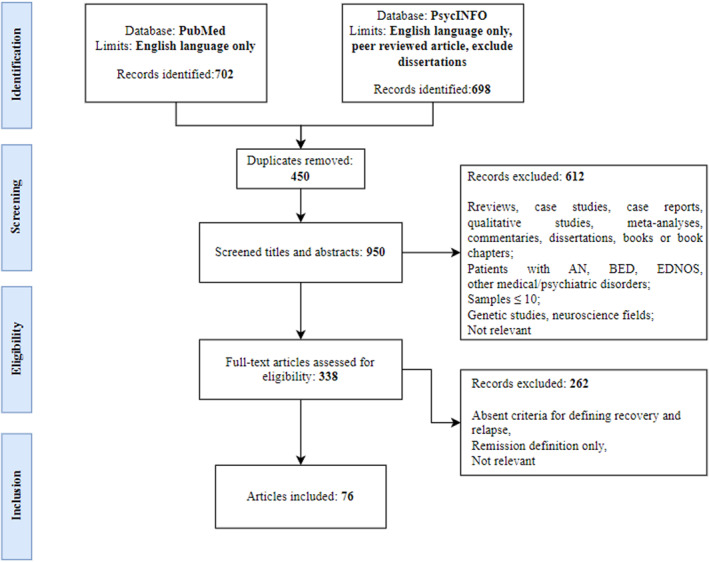
Flow chart of literature search and article selection process according to PRISMA criteria.

Figure 1 summarises literature search and article selection process.

### 2.2 Eligibility Criteria

The Population Intervention Comparison Outcomes Study framework (PICOS, Methley et al. 2014) was used in order to identify inclusion and exclusion criteria. For the purposes of this review, articles included provided a definition of recovery and/or relapse. A summary of the inclusion and exclusion criteria for article selection can be found in the table below (Table 1).

**TABLE 1:** PICOS table for inclusion/exclusion criteria.

PICOS	Inclusion criteria	Exclusion criteria
Patient	All age Female, male or mixed gender studies Patients with BN diagnoses	Patients without a formal diagnosis of BN, including subthreshold presentations of BN (e.g., OSFED‐BN type) Sample included other psychiatric diagnoses or medical illness not related to BN Samples with less than 10 subjects
Intervention	All types of psychological or medical interventions	
Comparison group	Studies with and without comparison groups	
Outcome	Definition of relapse and/or recovery Recovery and relapse rates in BN	Genetic or neurobiological alterations studies Studies on animal models Questionnaire validation studies Not relevant
Study design	Randomized controlled trials Longitudinal studies Retrospective studies Cross‐sectional studies	Case studies, case report Qualitative studies Reviews Meta‐analyses Books (or book chapters) Dissertations

Abbreviations: BN, bulimia nervosa; OSFED‐BN, Other Specified Feeding or Eating Disorder, bulimia nervosa‐type.

Moreover, since the focus of this systematic review was on the definitions of recovery and relapse in BN, which are typically established a priori, independently of sample composition, studies were included regardless of the proportion of BN participants.

### 2.3 Data Extraction

The data extraction process was conducted independently by two authors (*V.G. and F.P.*), considering the pre‐established PICOS criteria (see Table 1). The relevant data from each article were then systematically summarised in a separate table (*F.P.*). The data extracted included specific details about the study design, participants (sample size, age and gender), diagnosis, definition and rates of recovery, partial recovery and/or relapse (see Supporting Information S1: Table S1).

### 2.4 Quality and Risk of Bias Assessment

Studies included were then evaluated by two authors (*F.P. and E.T.*) using a customised checklist retrieved from the National Institutes of Mental Health's tool (2021). The tool evaluates multiple aspects of study quality, including research design, clarity of objectives, definition of the study population, adequacy of follow‐up, validity and reliability of outcome measures, etc. Each item was rated independently by two authors (*V.G. and F.P.*) and discrepancies in the evaluations were resolved through discussion until mutual agreement was reached. The ratings contributed to an overall methodological quality score for each study on a three‐level scale (i.e., strong, moderate or weak) (see Supporting Information S1: Table S2 and S3).

## 3 Results

### 3.1 Results of Literature Search

The search in Pubmed and PsycINFO yielded a total of 1400 articles. After removing 450 duplicates (32%), 612 articles (44%) were excluded based on title and abstract screening. Following a full‐text review of the remaining articles, 262 articles (19%) were excluded and 76 articles (5%) met the eligibility criteria and were included in the review.

Forty seven (61%) out of the selected studies provided definitions of recovery, 19 (25%) provided definitions of relapse, and 10 (13%) provided definitions of both recovery and relapse.

### 3.2 Characteristics of the Studies

Characteristics of the studies (e.g., diagnosis, age, gender, sample size, research design) included in the systematic review have been summarised in Supporting Information S1: Table S1.

Of the 76 studies included in the present systematic review, 49 (64%) were conducted on mixed ED samples, while 27 (36%) focused exclusively on BN.

In terms of sociodemographic characteristics of the samples used in the studies, the age range of participants was 12–65 years. Seventy‐five percent (*n* = 57) of the articles were carried out on young adults aged between 19.7 ± 4.8 and 29.9 ± 7.4 years, while 13% (*n* = 10) focused on adults aged between 30.4 ± 9.2 and 49.0 ± 6.1 years. Only 4% (*n* = 3) of the articles adopted a sample of adolescents aged between 15.8 ± 1.6 and 17.2 ± 2.1 years. The mean age of participants was not clearly stated in seven studies (9%).

Additionally, 71% (*n* = 54) of the studies had a sample consisting entirely of females, while the remaining studies (*n* = 15, 20%) used mixed samples but with a large percentage of female participants, ranging from 89.4% to 98.8%. The gender of participants was not specified in seven articles (9%).

Regarding research design, 72% (*n* = 55) adopted a longitudinal design with follow‐ups (FU). Of these, 29 (53%) authors used a FU period of one to five years, 16 (29%) studies used a FU of six to 10 years, and six (11%) articles used a period of less than 1 year. In addition, 5 (9%) authors followed participants for 17–22 years. One study (Eielsen et al. 2021) reported data at both 5 and 17 years of follow‐up and was therefore included in both categories.

Moreover, 13% (*n* = 10) of the studies used a cross‐sectional design, 10% (*n* = 7) used a randomized controlled trial (RCT) design, and 5% (*n* = 4) adopted a retrospective design.

Finally, different sample sizes were adopted. Specifically, 51% (*n* = 39) of the studies used a sample size of more than 150 participants, another 37% (*n* = 28) of articles had a sample size between 50 and 150 participants, while the remaining 12% (*n* = 9) included less than 50 participants.

### 3.3 Quality and Risk of Bias Assessment

In terms of quality, 46% (*n* = 35) articles were ranked as having moderate quality, 36% (*n* = 27) were ranked as weak, and 18% (*n* = 14) were ranked as having a strong quality (see Supporting Information S1: Table S3).

### 3.4 Definitions of Recovery

Out of the 76 reviewed articles, 47 (62%) provided definitions of recovery, while 10 (13%) included definitions for both recovery and relapse. Additionally, the remaining 19 studies (25%) included a definition of relapse only and will be discussed in following paragraphs (see Section 3.5).

Of the articles providing definitions of recovery, 33 (58%) gave a generalised definition of recovery for all EDs, including BN, and 24 (42%) defined recovery specifically for BN alone. Regarding specific criteria employed in the definitions of recovery adopted by the studies, 66,7% (*n* = 38) included definitions based on a single criterion, while 33% (*n* = 19) combined multiple criteria (a visual summary of the most common combinations of criteria used to define recovery is provided in Supporting Information S1: Figure S1). Specifically, 53% (*n* = 30) of the studies utilised diagnostic criteria, 51% (*n* = 29) used behavioural criteria, 30% (*n* = 17) used medical/physical criteria, and 18% (*n* = 10) used psychological criteria.

In 11 papers (14%) definitions of recovery from BN was also divided into partial and full recovery (Bardone‐Cone et al. 2016; Brewerton and Costin 2011; Cogley and Keel 2003; Eddy et al. 2007, 2008; Eielsen et al. 2021; Hergenroeder et al. 2015; Herzog, Sacks, et al. 1993; Herzog et al. 1996, 1999; Nakai et al. 2014), with partial recovery being defined as a phase characterised by a persistence of residual symptoms (e.g., behavioural or psychological), which were nevertheless less severe than those observed during the acute phase of the disorder.

Table 2 summarises specific criteria used by authors to define recovery and partial recovery in BN.

**TABLE 2:** Summary of definitions for recovery and partial recovery.

Criteria	Recovery (*n* = 57/76)					Partial recovery (*n* = 11/76)	
Diagnostic criteria	PSR ≤ 2 (*n* = 17, 57%)			Absence of a DSM diagnosis of an ED (*n* = 13, 43%)		PSR ≤ 4 (*n* = 5, 71%)	Reduction of symptoms to less than full DSM criteria (*n* = 2, 29%)
	***n* = 30, 53%**					***n* = 7, 64%**	
Behavioural criteria	Absence of binge‐eating or compensatory behaviours (*n* = 14, 48%)	Absence of binge‐eating, purging and restricting behaviours (*n* = 11, 38%)	Absence of binge‐eating, purging, restricting behaviours and excessive exercise (*n* = 3, 10%)		Binging and purging less than once a month (*n* = 1, 4%)	Reduction in binge‐eating and purging	
	***n* = 29, 51%**					***n* = 2, 18%**	
Medical/physical criteria	BMI ≥ 18.5 kg/m^2^ (*n* = 14, 82%)	BMI ≥ 18.5 kg/m^2^ and regular menstrual cycle (*n* = 5, 29%)			IBW > 90% and regular menstrual cycle (*n* = 3, 18%)	BMI ≥ 17.5 kg/m^2^	
	***n* = 17, 30%**					***n* = 1, 9%**	
Psychological criteria	EDE or EDE‐Q scores within 1 SD (*n* = 6, 60%)		Absence of psychological symptoms (e.g., no overconcern with body shape and weight) (*n* = 4, 40%)			Improved ED symptoms but persisting psychological symptoms (measured by EDE)	
	***n* = 10, 18%**					***n* = 3, 27%**	

Abbreviations: BMI, body mass index; DSM, Diagnostic and Statistical Manual of Mental Disorders; ED, Eating Disorder; EDE, Eating Disorder Examination; EDE‐Q, Eating Disorders Examination Questionnaire; IBW, Ideal Body Weight; PSR, Psychiatric Status Rating; SD, Standard Deviation.

The most prevalent criteria (*n* = 30, 53%) used in the definitions of recovery were DSM‐based *diagnostic criteria*. In particular, in several studies (*n* = 17, 57%) this was done using the Psychiatric Status Rating (PSR) score of ≤ 2 (Bloks et al. 2004; Clausen 2008; De Young et al. 2020; Eddy et al. 2007, 2008, 2017; Franko et al. 2005, 2008, 2018; Herzog et al. 1988; Herzog, Sacks, et al. 1993; Herzog et al. 1996, 1999; Keller et al. 1992; Keshishian et al. 2019; Murray et al. 2017; Shaw et al. 2012), which indicates the presence of residual symptoms without meeting diagnostic criteria. The PSR is a six‐point ordinal scale used to assess the presence or absence of symptoms, on a weekly basis, and is derived from the Longitudinal Interval Follow‐up Evaluation (LIFE) or similar semi‐structured interviews (Keller et al. 1987). In the studies, ratings were assigned by trained clinicians or research staff, usually based on weekly assessments of ED symptomatology. A score of 1 indicates the absence of BN symptoms or attitudes. A score of 2 indicates the presence of residual symptoms without meeting diagnostic criteria, while 3 points correspond to mild symptoms accompanied by functional impairment. At score 4, marked symptoms are observed in the absence of all diagnostic criteria, whereas scores of 5 or 6 indicate a severe diagnosis of BN. Eighty‐two percent (*n* = 14) of these authors required criteria to be met over a period of at least two consecutive months in order to define recovery (Bloks et al. 2004; Franko et al. 2005, 2008; Herzog et al. 1988; Herzog, Sacks, et al. 1993; Herzog et al. 1996, 1999; Keller et al. 1992; Shaw et al. 2012) to 1 year (De Young et al. 2020; Eddy et al. 2017; Franko et al. 2018; Keshishian et al. 2019; Murray et al. 2017).

Other studies (*n* = 13, 43%) required the absence of a clinical diagnosis of EDs according to structured or semi‐structured clinical interviews based on DSM‐IV/DSM‐IV‐TR (Bardone‐Cone et al. 2016; Castellini et al. 2011, 2012, 2014; Hergenroeder et al. 2015; Jacobi et al. 2017; Kuipers et al. 2017; Levallius et al. 2016; Rossotto et al. 1996; Stice et al. 2009; Yu et al. 2013) or DSM‐5 (Cabelguen et al. 2023; Castellini et al. 2017) criteria. Fifty‐four percent (*n* = 7) of these articles also specified a period of absence from diagnosis that was required to define recovery. This period of absence ranged from 1 month to 3 months in 86% (*n* = 6) of articles (Bardone‐Cone et al. 2016; Castellini et al. 2014; Hergenroeder et al. 2015; Levallius et al. 2016; Stice et al. 2009; Yu et al. 2013), while one article required an abstinence period of 1 year (Rossotto et al. 1996).

A significant number of studies (*n* = 29, 51%) used *behavioural criteria* to define recovery, with 14 (48%) requiring cessation of binge eating, and purging behaviours (Bailer et al. 2004; Brewerton and Costin 2011; Cogley and Keel 2003; Eielsen et al. 2021; Field et al. 1997; Garte et al. 2015; Hsu and Sobkiewicz 1989; Keski‐Rahkonen et al. 2009, 2012; Klump et al. 2004; Larrañaga et al. 2014; Lock et al. 2013; Mitchell et al. 2011; Yu et al. 2013). In addition to these symptoms, 11 (38%) also required the absence of weight loss associated with laxative abuse, fasting, or food restriction (Bardone‐Cone et al. 2016; Forney et al. 2022; Harrison et al. 2011, 2014; Kordy et al. 2002; Nakai et al. 2014; Reas et al. 2000; Richard et al. 2005; Stein et al. 2002; Von Holle et al. 2008; von Ranson et al. 1999), while three (10%) added also the absence of excessive exercise (Melisse et al. 2022; Silén et al. 2021; Wagner et al. 2006). A minimum abstinence period needed to define recovery was added in 83% (*n* = 24) of these studies. Fifty‐eight percent (*n* = 14) of these required at least 1 year of abstinence (Field et al. 1997; Harrison et al. 2011, 2014; Keski‐Rahkonen et al. 2009, 2012; Klump et al. 2004; Kordy et al. 2002; Richard et al. 2005; Silén et al. 2021; Stein et al. 2002; Von Holle et al. 2008; von Ranson et al. 1999; Wagner et al. 2006), while 42% (*n* = 10) required it to be at least one to 6 months long (Bailer et al. 2004; Bardone‐Cone et al. 2016; Cogley and Keel 2003; Eielsen et al. 2021; Forney et al. 2022; Hsu and Sobkiewicz 1989; Melisse et al. 2022; Mitchell et al. 2011; Nakai et al. 2014; Yu et al. 2013). Moreover, one article (4%) defined recovery as engaging in binging and purging behaviours less than once a month for the past 3 months (Herzog et al. 1993). Behavioural symptoms were assessed using non‐structured (*n* = 9, 31%) and semi‐structured interviews (*n* = 20, 69%), such as the Eating Disorder Examination (EDE) and the Eating Disorder Examination‐Questionnaire (EDE‐Q).

Seventeen reviewed studies (30%) applied *medical/physical criteria* to define recovery. Body mass index (BMI ≥ 18.5 kg/m^2^) was the main criterion used (*n* = 14, 82%) (Bardone‐Cone et al. 2016; Eielsen et al. 2021; Forney et al. 2022; Garte et al. 2015; Keski‐Rahkonen et al. 2009, 2012; Melisse et al. 2022; Silén et al. 2021; Yu et al. 2013). Although underweight status is not typically associated with BN, the BMI criterion in BN samples was adopted not to define weight restoration per se, but rather to ensure that individuals with current or past subthreshold AN were not erroneously included in the recovered BN group (Keski‐Rahkonen et al. 2009, 2012). Similarly, a regular menstrual cycle was also used as criterion in five articles (Harrison et al. 2011, 2014; Nakai et al. 2014; Stein et al. 2002; von Ranson et al. 1999). Other authors (*n* = 3, 18%) combined regular menstruation with ideal body weight (IBW > 90%) (Herzog et al. 1993; Lock et al. 2013; Wagner et al. 2006).

*Psychological criteria* to define recovery were used in fewer articles (*n* = 10, 18%). Most authors (*n* = 6, 60%) used the EDE or the EDE‐Q to define psychological recovery, requiring a global score within 1 SD of the normative reference sample (Eielsen et al. 2021; Forney et al. 2022; Garte et al. 2015; Harrison et al. 2011, 2014; Lock et al. 2013; Melisse et al. 2022; Yu et al. 2013) or all four subscale scores within 1 SD of the normative reference sample meet this criterion (Bardone‐Cone et al. 2016; Forney et al. 2022). When specified, normative reference samples were generally drawn from age‐matched community populations (Bardone‐Cone et al. 2016; Forney et al. 2022; Garte et al. 2015), although most studies did not report whether gender identity or other demographic variables were considered in norm derivation.

The remaining authors (*n* = 4, 40%) measured lack of excessive preoccupations with body shape and weight using other instruments (e.g., EAT‐26) (Herzog et al. 1993; Larrañaga et al. 2014; Nakai et al. 2014; Silén et al. 2021). Eight (80%) articles included a time requirement, stating that the absence of psychological symptoms occurred for at least 1 month (25%, *n* = 2) (Bardone‐Cone et al. 2016; Eielsen et al. 2021), 3 months (50%, *n* = 4) (Cogley and Keel 2003; Forney et al. 2022; Herzog et al. 1993; Nakai et al. 2014) and 1 year (25%, *n* = 2) (Larrañaga et al. 2014; Silén et al. 2021).

### 3.5 Definition of Relapse

Among the 76 reviewed articles, 19 (25%) included definitions of relapse, while 10 (13%) offered definition for both recovery and relapse. Specifically, 45% (*n* = 13) of studies provided a definition of relapse for EDs, including BN, and 55% (*n* = 16) gave a definition of relapse for BN. In terms of the criteria used to define relapse, 15 (52%) articles used diagnostic criteria, and 14 (48%) studies used behavioural criteria.

Table 3 summarises the criteria used by authors to define relapse in BN.

**TABLE 3:** Summary of definitions for relapse.

Criteria	Relapse (*n* = 29/76)		
Diagnostic criteria	PSR ≥ 5 (*n* = 2, 13%)	Currently meeting diagnostic criteria for ED (*n* = 11, 74%)	
	***n* = 15, 52%**		
Behavioural criteria	Mean of 1 episode of binge‐eating and/or purging for week (*n* = 5, 36%)	Mean of 2 episodes of binge‐eating and/or purging for week (*n* = 7, 50%)	Vomiting or binging to be more frequent than the patient's baseline (*n* = 2, 14%)
	***n* = 14, 48%**		
Medical/physical criteria	ꟷ		
Psychological criteria	ꟷ		

Abbreviations: ED, Eating Disorder; PSR, Psychiatric Status Rating.

Relapse is defined in 52% (*n* = 15) of the articles using *diagnostic criteria.* Seventy‐four percent (*n* = 11) of studies defined relapse as a change from DSM‐IV partial or full remission to full syndrome EDs (Bergh et al. 2002, 2013; Castellini et al. 2011; Clausen 2008; Fairburn et al. 2000; Grilo et al. 2012; Kordy et al. 2002; Larrañaga et al. 2014; Richard et al. 2005; Stice et al. 2009; Yu et al. 2013). Of these, three authors required at least 2 months (67%, *n* = 2) (Grilo et al. 2012; Herzog et al. 1999) or 3 months (33%, *n* = 1) for symptoms recurrence (Clausen 2008). Instead, two (13%) authors required full DSM‐III criteria to be met for at least two consecutive weeks, after a period of recovery or remission (Commerford et al. 1997; Keller et al. 1992). In addition, two (13%) articles defined relapse as a PSR score of ≥ 5 (Herzog et al. 1999; Keel et al. 2005).

*Behavioural criteria* are also used to define relapse (*n* = 14, 48%). 12 (86%) authors required compensatory behaviours (e.g., vomiting and using laxatives) and/or binge eating at least once or twice a week per month after a period of recovery or remission (Bohon et al. 2009; Fairburn et al. 1993; Field et al. 1997; MacDonald et al. 2015; McFarlane et al. 2008; Mitchell et al. 1985; Olmsted et al. 1994, 1996, 2005, 2015; Pyle et al. 1990; Sollid et al. 2021). A temporal duration was added in 71% (*n* = 10) of these *n* = 14 articles, where the behavioural criteria must be met for one month (30%, *n* = 3) (Field et al. 1997; Pyle et al. 1990; Sollid et al. 2021), two months (10%, *n* = 1) (Mitchell et al. 1985), or three consecutive months (60%, *n* = 6) (MacDonald et al. 2015; McFarlane et al. 2008; Olmsted et al. 1994, 1996, 2005, 2015).

### 3.6 Rates of Recovery and Relapse

Out of the studies reviewed, 50% (*n* = 38) reported overall recovery rates for BN ranging from 9.1% to 91%. Table 4 summarises recovery rates according to follow‐up duration. Despite substantial variation across studies in terms of criteria used and rates found, recovery rates tend to increase over longer follow‐up periods after patients received treatment.

**TABLE 4:** Recovery rates in bulimia nervosa reported by included studies, grouped by follow‐up duration.

Recovery rates												
Follow‐up (years)												
Articles	> 1	1–1.5	2–2.5	3–3.5	4	5	6	7	8	9–10	17–22	Criteria
Melisse et al. (2022)	9.1% (*n* = 34)											B + P
Hergenroeder et al. (2015)	28% (*n* = 8)											D
Herzog et al. (1988)	33% (*n* = 10)											D
Bailer et al. (2004)		21% (*n* = 10)										B
Mitchell et al. (2011)		21.8% (*n* = 51)										B
Field et al. (1997)		37.7% (*n* = 40)										B
Yu et al. (2013)		16.7%–40.6%										B + D + PSY + P
Jacobi et al. (2017)		50.6% (*n* = 83)										D
Herzog et al. (1993)		56% (*n* = 53)										D
Castellini et al. (2017)		57.5% (*n* = 23)										D
Kuipers et al. (2017)		75% (*n* = 3)										D
Kordy et al. (2002)			16% (*n* = 68)									B
Clausen (2008)			36.7% (*n* = 11)									D
Herzog et al. (1999)			53% (*n* = 58)									D
Richard et al. (2005)			74% (*n* = 313)									B
Herzog et al. (1993)				20% (*n* = 3)								B + PSY + P
Castellini et al. (2012)				40% (*n* = 34)								D
Larrañaga et al. (2014)				42.1% (*n* = 8)								B + PSY
Castellini et al. (2017)				60% (*n* = 24)								D
Keller et al. (1992)				69% (*n* = 21)								D
Herzog et al. (1996)					56.2% (*n* = 86)							D
Hsu and Sobkiewicz (1989)					60% (*n* = 21)							B
Brewerton and Costin (2011)					61% (*n* = 32)							B
Silén et al. (2021)						23.1% (*n* = 4)						B + PSY + P
Keski‐Rahkonen et al. (2009)						55% (*n* = 55)						B + P
Castellini et al. (2011)							49.6% (*n* = 68)					D
Castellini et al. (2014)							49.6% (*n* = 68)					D
Nakai et al. (2014)								46% (*n* = 39)				B + PSY + P
Eddy et al. (2008)								65.6% (*n* = 84)				D
Herzog et al. (1999)								73% (*n* = 80)				D
Shaw et al. (2012)									79.1% (*n* = 87)			D
Stice et al. (2009)									91% (*n* = 29)			D
Forney et al. (2022)										29% (*n* = 38)		B + PSY + P
De Young et al. (2020)										30.2% (*n* = 33)		D
Eddy et al. (2007)										62.5% (*n* = 110)		D
Eddy et al. (2017)										68.2% (*n* = 75)		D
Reas et al. (2000)										72.7% (*n* = 32)		B
Von Holle et al. (2008)											19% (*n* = 52)	B
Murray et al. (2017)											49% (*n* = 52)	D
Franko et al. (2018)											68.2% (*n* = 52)	D
Eddy et al. (2017)											68.2% (*n* = 75)	D

Abbreviations: B, behavioural criteria; D, diagnostic criteria; P, physical criteria; PSY, psychological criteria.

Twenty three (30%) articles instead reported relapse rates between 16% and 63% overall. Table 5 presents relapse rates stratified by follow‐up length. Overall, data suggest that, despite the studies showing considerable variability across relapse rates, they tend to be higher in the early years following treatment (> 1–2.5 years).

**TABLE 5:** Relapse rates in bulimia nervosa reported by included studies, grouped by follow‐up duration.

Relapse rates												
Follow‐up (years)												
Articles	> 1	1–1.5	2–2.5	3–3.5	4	5	6	7	8	9–10	22	Criteria
Field et al. (1997)	25% (*n* = 26)											B
MacDonald et al. (2015)	37.6% (*n* = 38)											B
Mitchell et al. (1985)	40% (*n* = 12)											B
Olmsted et al. (2015)	27.6% (*n* = 32)											B
Pyle et al. (1990)	30% (*n* = 18)											B
Romano et al. (2002)	39.3% (*n* = 59)											B
Sollid et al. (2021)	28.6% (*n* = 8)											B
Walsh et al. (1991)	29% (*n* = 6)											B
Fairburn et al. (1993)		16% (*n* = 12)										B
MacDonald et al. (2015)		51.1% (*n* = 46)										B
Olmsted et al. (2005)		21%–55%										B
Yu et al. (2013)		8.3%–60%										B + D
McFarlane et al. (2008)			28% (*n* = 5)									B
Olmsted et al. (1994)			31.3% (*n* = 15)									B
Olmsted et al. (1996)			24% (*n* = 40)									B
Richard et al. (2005)			37.4% (*n* = 158)									D
Fairburn et al. (2000)				32% (*n* = 8)								D
Fairburn et al. (2000)					33% (*n* = 9)							D
Fairburn et al. (2000)						26% (*n* = 8)						D
Castellini et al. (2011)							17.7% (*n* = 12)					D
Grilo et al. (2012)							46% (*n* = 16)					D
Herzog et al. (1999)								35.3% (*n* = 39)				D
Stice et al. (2009)									41% (*n* = 13)			D
Keel et al. (2005)										35% (*n* = 38)		D
Eddy et al. (2017)											20.5% (*n* = 15)	D

Abbreviations: B, behavioural criteria; D, diagnostic criteria.

## 4 Discussion

The purpose of this review was to identify and summarise criteria to define recovery and relapse in BN, in order to achieve a better consensual definition of these stages of illness to facilitate the longitudinal assessment of this ED. The present findings, however, confirm a lack of agreement among researchers on the definitions of both these stages of illness.

The majority of the authors used the *diagnostic criteria* to define recovery from BN, meaning that recovery could be defined in the absence of a DSM‐IV or DSM‐5 diagnosis of BN. A significant proportion of authors instead preferred to use a *behavioural criteria* to define recovery, which required the absence of two or more core symptoms of the disorder, such as binge eating episodes and compensatory behaviours including self‐induced vomiting, abuse of laxatives or diuretics, fasting and excessive exercise. A *medical/physical criteria*, which is defined as a healthy weight range and is primarily measured by BMI (≥ 18.5 kg/m^2^), was also discussed in fewer articles. Although a BMI threshold is generally more relevant to anorexia nervosa, several authors adopted it to explicitly exclude individuals with current or past underweight status or subclinical AN (Keski‐Rahkonen et al. 2009, 2012). In many cases, this criterion was adopted in studies using transdiagnostic or mixed samples, where the risk of diagnostic overlap was higher.

Finally, *psychological criteria* related to recovery, such as reduced obsession with food, weight and body shape, were not sufficiently assessed in the research. Some authors report the time required for different criteria to describe recovery from BN, although there is currently no consensus between studies. These temporal criteria across definitions vary widely, from 1 month to 1 year.

It is often argued by a number of authors that the psychological component of EDs, which may include thoughts about the body, food, and eating, is not adequately represented in the concept of recovery (Bardone‐Cone et al. 2018; Williams et al. 2012). Instead, there is a predominant emphasis on the absence of diagnostic and behavioural symptoms. Several studies included in this review have found that individuals who were abstinent from behavioural symptoms at FU may still experience significant psychological symptoms over time. These include body dissatisfaction, low self‐esteem, anxiety and depressive symptoms (Bardone‐Cone et al. 2016; Cogley and Keel 2003; Keski‐Rahkonen et al. 2009; Stein et al. 2002). This is in line with recent research (Bardone‐Cone et al. 2018; Monteleone and Cascino 2021; Robinson et al. 2024) highlighting that overvaluation of body shape and weight, along with cognitive restraint, represent a central psychopathological feature that significantly affect the course and outcome of ED recovery. Transdiagnostic factors such as feelings of ineffectiveness, low interoceptive awareness, and difficulties in affect regulation also seem to play a crucial role in the maintenance and development of EDs according to a recent network analysis (Monteleone and Cascino 2021). Therefore, a deeper understanding of both ED‐specific and non‐ED specific psychological factors is essential for clinicians to develop and tailor effective interventions and accurate recovery criteria.

A significant heterogeneity emerged in criteria and duration required to define recovery in the reviewed studies, with some using only diagnostic or behavioural criteria, and others including psychological or physical aspects (Bardone‐Cone et al. 2018; Gorrell et al. 2020). As a result, reported rates of recovery were difficult to compare across studies and the interpretation of findings is limited. According to longitudinal studies on mixed ED samples (Bardone‐Cone et al. 2019) as well, recovery should be considered a multidimensional outcome, but the lack of disorder‐specific data and shared criteria to define outcomes, particularly for BN, limits the ability to analyse recovery trajectories specific to this disorder.

In the studies included in this systematic review (Yu et al. 2013), lower recovery rates at the end of treatment (EOT) emerged when recovery was defined by abstinence from compensatory and binge behaviours compared with definitions based on the absence of diagnostic criteria. However, these findings changed at FU, where recovery rates increased when abstinence from behavioural symptoms was used as a recovery criterion (Yu et al. 2013). The use of different time criteria also affected recovery rates. For example, requiring shorter periods of abstinence from compensatory and binge behaviours (e.g., 6 months) was associated with higher recovery rates (De Young et al. 2020). On the other hand, requiring recovery criteria to be met for at least 18 months led to lower recovery rates, but was also correlated with less recurrence of eating symptoms over time (De Young et al. 2020). FU length also impacted recovery rates, with longer FU generally being associated with higher recovery rates, despite high variability among the studies. These results are consistent with those of a recent meta‐analysis (Solmi et al. 2024), which observed that longer illness duration and follow‐up periods were associated with both increased chances of recovery and reduced chronicity, especially when more rigorous or sustained definitions were applied. Indeed, findings from this review suggest that sustained recovery is most frequently defined using a follow‐up period and/or a symptom‐free duration of at least 1 year, particularly when multiple domains are considered (De Young et al. 2020; Field et al. 1997). However, the required time criterion varies across studies and the reliability of retrospective data over extended periods, especially regarding cognitive symptoms of EDs, may be limited and highly dependent on the method of assessment and criteria considered. This highlights the importance of considering both how and when recovery is assessed when interpreting outcome data in BN, and further supports the need for standardised, time‐sensitive criteria to evaluate recovery trajectories.

Finally, relapse is defined as the return of eating symptoms after a period of symptom remission (Miskovic‐Wheatley et al. 2023). According to the results, relapse was mostly defined using *diagnostic criteria,* specifically as the transition from partial or complete remission to full syndrome EDs according to the DSM‐III‐R or DSM‐IV. *Behavioural criteria*, were also proposed by a number of authors, and relapse was defined as the return of binge eating and compensatory behaviours after a period of recovery. Some articles also state that the persistence of symptoms must be observed for two to 3 months. However, neither physical nor psychological criteria were employed to define relapse.

The lack of assessment of the psychological symptoms related to relapse may lead to inadequate recognition of the pervasiveness of these symptoms and their role in exacerbating the disorder. Residual symptoms have been shown to increase the risk of relapse (Fairburn et al. 1993; Keel et al. 2005; Olmsted et al. 1994; Tomba et al. 2019). In particular, overestimation of weight and shape, fear of gaining weight and feeling fat, may promote the development of compensatory weight control behaviours and a new illness onset (Stice et al. 2021). Therefore, understanding the social, biological and individual risk factors that may influence the progression of the disorder from one stage to the next is a crucial aspect of preventing its progression (McGorry et al. 2006).

When considering BN relapse rates, data partially suggest that, similarly to what has been observed in the literature, relapse is a common event following recovery, with the period of greatest vulnerability occurring in the first few years after symptom remission (Miskovic‐Wheatley et al. 2023), with rates stabilising after 2 years (Field et al. 1997).

Olmsted and colleagues (2005) also observed a higher incidence of relapse in patients who reported a greater number of symptoms at the EOT than in those who were completely symptom free. Furthermore, using stricter relapse criteria over longer periods (3 months vs. one month) was shown to reduce the incidence of relapse (Olmsted et al. 2005). However, the high heterogeneity in the studies hinders interpretation and generalisability of finding for this outcome and a clear and consistent difference in relapse rates across follow‐up durations did not emerge from the data.

## 5 Conclusion

The present review underlines the complexity of defining recovery and relapse in bulimia nervosa (BN), reflecting a lack of consensus among researchers and a wide range of criteria used. While diagnostic, behavioural and physical criteria are commonly used to assess recovery and relapse, psychological factors are often inadequately represented, despite articles showing that residual psychological symptoms may persist even after behavioural recovery and increase the risk of relapse (Tomba et al. 2019). Moreover, there is a lack of standardised temporal criteria for defining these phases in BN and even when time frames are provided they are not consistent across studies.

However, despite the considerable heterogeneity observed across studies, this review highlights a set of criteria for recovery and relapse that are most consistently endorsed in the literature. For *recovery*, definitions most commonly combined a *diagnostic criterion*, usually operationalised through a PSR score ≤ 2, with a *behavioural criterion*, defined as abstinence from binge eating and compensatory behaviours, most often assessed with the EDE or EDE‐Q. Some studies additionally included a *medical/physical criterion*, such as maintaining a BMI ≥ 18.5. Although used less frequently in the definitions found, a *psychological criterion*, such as EDE or EDE‐Q global and subscale scores within one standard deviation of community norms, should also be used to capture residual cognitive and affective symptoms that may persist beyond behavioural remission and increase vulnerability to relapse. Therefore, incorporating *psychological criteria* may also help to better differentiate between partial and full recovery.

For relapse, the most common definition adopted *behavioural criteria,* such as the re‐emergence of binge eating and/or compensatory behaviours after a period of remission, sometimes combined with a *diagnostic criterion,* that is, meeting again the DSM diagnostic thresholds for BN after a phase of partial or full recovery, with persistence of symptoms over at least one to 3 months.

From a clinical and research perspective, incorporating these criteria into multidimensional conceptualizations of recovery and relapse may enhance the comparability of research findings and promote the development of shared, standardized definitions that can be consistently applied across clinical contexts.

Finally, while the present systematic review focused primarily on criteria for recovery and relapse in BN defined by clinicians, qualitative research (Lindgren et al. 2015) suggests that individuals with BN may define recovery in ways that differ from standard clinical outcomes. Since qualitative research on the topic is still lacking, future research should explore patients' perspectives on recovery and relapse more systematically to incorporate patient‐defined criteria into more comprehensive definitions of recovery and relapse in BN.

The results of this review should also be considered according to several limitations. Only two databases (PubMed and PsycINFO) were used for literature search, potentially excluding articles only available on other databases. Moreover, the quality of included articles was moderate or low for most articles, reducing the robustness of the findings, mainly due to unclear outcome definitions in the literature. Studies also relied on different diagnostic systems (e.g., DSM‐III, DSM‐IV, DSM‐5), which complicated comparisons and may have influenced how recovery and relapse were operationalised. Similarly, many studies used mixed samples and adopted transdiagnostic definitions, complicating the generalisation of results to BN only.

## Author Contributions

Conceptualization: E.T. and V.G.; Data curation: F.P. and V.G.; Methodology: V.G.; Project administration: E.T.; Supervision: E.T.; Validation: E.T.; Visualization: F.P.; Writing – original draft: F.P.; Writing – review and editing: V.G. and E.T.

## Conflicts of Interest

The authors declare no conflicts of interest.

## Supporting information


Supporting Information S1

## Data Availability

Data sharing not applicable to this article as no datasets were generated or analysed during the current study.
