# Supplementary material for: Defining Recovery and Relapse in Bulimia Nervosa: A Systematic Review of the Literature
Source: Eur Eat Disord Rev. 2025 Sep 24;34(2):358–74. doi: 10.1002/erv.70033 (PMC12862549; doi:10.1002/erv.70033)
Supplement: Supplementary file 1 — Supporting Information S1 [file ERV-34-358-s001.docx]

SUPPLEMENTARY MATERIAL

**Supplementary Table S1.** *Characteristics and outcome of selected studies*

| Article | Sample size (%F)  Age ± SD (range) | Sample type | Study type | Assessment diagnosis | Definitions of recovery, partial recovery, relapse | Rates |
| --- | --- | --- | --- | --- | --- | --- |
| **Articles giving definitions of recovery and partial recovery** | | | | | | |
| Bailer, de Zwaan, Leisch et al.  (2004) | n=81  (n/a %)  M= 23.7 ± 4.5 | BN | RCT  self-help vs CBT  FU= 1 year | DSM-IV | Recovery: abstinent from binge eating or purging for one month. | Recovery rates at FU: 21% (n=10/48) |
| Bardone-  Cone, Butler, Balk et al.  (2016) | n=155  (100%)  M_ED_=21.8 ± 4.3  (16-40 years) | BN=4  RBN=11  AN=9  RAN=21  EDNOS=33  REDNOS=10  C=67 | Cross-sectional study  active ED vs PR-ED vs FR-ED | DSM-IV | Full recovery ED: 1) absence of an ED diagnosis according to the DSM-IV; 2) BMI≥18,5 kg/m^2^; 3) no binge eating, purging, or fasting in the past 3 months; 4) score on the EDE-Q within 1 SD of the normative values of the reference population, over the past 4 weeks.  Partial recovery ED: met all the criteria of full recovery except for psychological recovery. |  |
| Bloks, Hoek, Callewaert et al.  (2004) | n=146  (100%)  M_ED_=27.5 ± 8.1 | BN=47  AN=72  EDNOS=27 | Longitudinal  study  FU=2.5 years | DSM–IV | Recovery BN: PSR ≤2 for at least 2 months. | Recovery rates ED at FU: 36% (n=50) |
| Brewerton & Costin  (2011) | n=118  (n/a%)  M_BN_=29.9 ± 7.4  (22-57 years) | BN=52  AN=66 | Longitudinal  study  FU= 10 years | DSM-IV | Full recovery BN: complete cessation of binge eating and purging behaviors.  Partial recovery BN: at least a 50% reduction in binge eating and purging. | Partial recovery rates BN at FU (4 years): 14% (n=7)  Full recovery rates BN at FU (4 years): 61% (n=32) |
| Cabelguen, Saillard, Vanier et al.  (2023) | n=186  (96%)  M_ED_= 26.5 ± 9.9 | BN=100  BED=32  AN=54 | Longitudinal  study  FU=1 year | DSM-5 | Recovery ED: absence of any eating disorders at FU. | Recovery rates ED at FU: 20% (n=38) |
| Castellini,  Montanelli, Faravelli, et al.  (2014) | n=564  (n/a%)  M_ED_=n/a  (18-60 years) | BN=137  BED=262  AN=165 | Longitudinal  study  FU=6 years | DSM-IV | Recovery ED: not fulfill the DSM-IV criteria for any eating disorder (including EDNOS) for at least 8 weeks. | Recovery rates BN at FU: 49.6% (n=68) |
| Castellini, Lelli, Corsi et al.  (2017) | n=79  (100%)  M_BN_= 30.4 ± 9.2  (18-45 years) | BN=40  AN=39 | Longitudinal  study  FU=3 years | DSM-5 | Recovery ED: not fulfill the DSM-5 criteria for any ED (including OSFED). | Recovery rates BN at FU (1 year): 57.5% (n=23)  Recovery rates BN at FU (3 years): 60% (n=24) |
| Castellini, Mannucci, Lo Sauro et al.  (2012) | n=218  (BN=96.5%)  M_BN_=29.5 ± 9.1  (18-60 years) | BN=85  BED=133 | Longitudinal  study  FU=3 years | DSM-IV-TR | Recovery ED: not fulfill the DSM-IV criteria for any eating disorder (including EDNOS). | Recovery rates BN at PT: 31% (n=26)  Recovery rates BN at FU: 40% (n=34) |
| Cogley & Keel  (2003) | n=118  (100%)  M_FR_ = 34.7 ± 10.2  M_PR_= 33.1 ± 8.3  (18-65 years) | FR-BN= 31  PR-BN=28  C=59 | Cross-sectional study  FR-BN vs PR-BN vs C | DSM–IV | Full recovery: 1) no binge eating episodes and inappropriate compensatory behavior, 2) scores ≤ of 3 on the influence of weight and the influence of shape items of the EDE; for the past 3 months.  Partial recovery: 1) no behavioral symptoms for the past 3 months.; 2) score ≥ of 4 on the influence of weight and the influence of shape items of the EDE. |  |
| De Young, Kambanis, Bottera et al.  (2020) | n=246  (100%)  M_ED_=24.8 ± 6.7 | BN=110  AN=136 | Longitudinal  study  FU=9.5 years | DSM-IV | Recovery BN: PSR≤2 for 6 months. | Recovery rates BN at FU: 30.2% (n=33)  As the duration criterion increased from 27 to 78 weeks, the recovery rate significantly decreased from 30.2% to 23.7%, and the symptom return rate significantly decreased from 14.6% to 11.7% |
| Eddy, Dorer, Franko et al.  (2008) | n=216  (100%)  M_BN_=25.2 ± 6.3 | BN=128  AN=88 | Longitudinal  study  FU=7 years | DSM-IV-TR | Full recovery ED: PSR≤2.  Partial recovery ED: PSR=3 o 4. | Partial recovery rates BN at FU: 82.8% (n=106)  Full recovery rates BN at FU: 65.6% (n=84) |
| Eddy, Dorer, Franko et al.,  (2007) | n=176  (100%)  M=n/a | BN | Longitudinal  study  FU=9 years | DSM-IV | Full recovery: PSR ≤ 2  Partial recovery: PSR≤ 4 (EDNOS) | Partial recovery rates at FU: 84.3% (n=151)  Full recovery rates at FU: 62.5% (n=110) |
| Eddy, Tabri, Thomas et al.  (2017) | n=176  (100%)  M_BN_= 49.0 ± 6.1  (means at 22 years FU) | BN=76  AN=100 | Longitudinal  study  FU= 22 years | DSM-IV | Recovery ED: PSR ≤ 2 for 52 consecutive weeks. | Recovery rates BN at FU (9 years): 68.2% (n=75/110)  Recovery rates BN at FU (22 years): 68.2% (n=75/110)  Relapse rates BN at FU (22 years): 20.5% (n=15) |
| Eielsen, Vrabel, Hoffart et al.  (2021) | n=86  (98.8%)  M_BN_=30.6±7.0 | BN=25  AN=23  OSFED=14 | Longitudinal  study  FU= 5 - 17 years | DSM-5 | Full recovery ED: 1) No binges or compensatory behavior (e.g., vomiting, laxative use) the previous 12 weeks; 2) BMI ≥18.5 kg/m^2^; 3) Global EDE within 1 SD of healthy controls the previous month.  Partial recovery ED: 1) Binges and/or weight compensatory behavior less than weekly the previous 12 weeks; 2) BMI ≥17.5 kg/m^2^; 3) Global EDE within 2 SD of healthy controls the previous month. | Partial recovery rates ED at FU (5 years): 14.5% (n=9)  Full recovery rates ED at FU (5 years): 29% (n=18)  Partial recovery rates ED at FU (17 years): 21% (n=13)  Full recovery rates ED at FU (17 years): 29% (n=18) |
| Forney, Brown, Crosby et al.,  (2022) | n=217  (100%)  M_ED_=22.8 ± 5.3  (18–43 years) | BN=133  PD=84 | Longitudinal  study  FU= 10.6 ± 3.7 years | DSM-5 | Recovery ED: 1) BMI≥18,5 kg/m^2^; 2) abstinence from binge-eating, purging, and fasting over the prior 12 weeks on the EDE; 3) EDE-Q global score were within 1 SD of community norms for their age. | Recovery rates BN at FU: 29% (n=38) |
| Franko, Dorer, Keel et al.  (2008) | n=246  (100%)  M_ED_=24.6 ± 6.7 | BN=110  AN=136 | Longitudinal  study  FU=8.6 years | DSM-IV | Recovery ED: PSR ≤ 2 for 8 consecutive weeks. |  |
| Franko, Dorer, Keel et al.,  (2005) | n=246  (100%)  M_ED_=24.6 ± 6.7 | BN=110  AN=136 | Longitudinal  study  FU=8.6 years | DSM-IV | Recovery ED: PSR ≤ 2 for 8 consecutive weeks. |  |
| Franko, Tabri, Keshaviaha et al.  (2018) | n=176  (100%)  M_ED_=24.6 ± 6.7  (13-45 years) | BN=76  AN=100 | Longitudinal  study  FU= 22 years | DSM-IV | Recovery ED: PSR ≤ 2 for 52 consecutive weeks. | Recovery rates BN at FU: 68.2% (n=52) |
| Garte, Hagen, Reas et al.  (2015) | n=62  (93.5%)  M_ED_=27.6 ± 7.2  (18-47 years) | BN=29  AN=13  EDNOS=20 | Longitudinal  study  FU=16-18 weeks | DSM-IV | Recovery ED: 1) BMI≥ 18.5 kg/m^2^; 2) EDE-Q +1 SD above community mean. | Recovery rates BN at PT: 47.6% (n=10/21) |
| Harrison,  Mountford & Tchanturia  (2014) | n=317  (100%)  M_BN_= 27.9 ± 9.5  (18-55 years) | BN=46  AN=105  RAN=30  C=136 | Cross-sectional study  ED vs C | DSM-IV | Recovery ED: 1) BMI>18.5 kg/m^2^ and have regular menstrual cycles; 2) absence of ED behaviours such as restriction or binge-purge symptoms measured by the EDE-Q; criteria met for at least 1 year. |  |
| Harrison, Treasure & Smillie  (2011) | n=286  (100 %)  M_ED_=34.2 ±10.5  (18-55 years) | BN=34  AN=84  RED=74  HC=91 | Cross-sectional study  ED vs C | DSM-IV | Recovery ED: 1) BMI>18.5 kg/m^2^ and have regular menstrual cycles; 2) absence of ED behaviours such as restriction or binge-purge symptoms measured by the EDE-Q; criteria met for at least 1 year. |  |
| Hergenroeder, Wiemann, Henges et al.  (2015) | n=218  (BN=97%)  M_BN_=17.2 ± 2.1  (10-25 years) | BN=29  AN=88  EDNOS=  101 | Retrospective study | DSM-IV | Full recovery BN: no criterion symptoms for the previous 8 weeks.  Partial recovery BN: improvement in some criterion symptoms, yet some persisted in the previous 8 weeks. | Partial recovery rates BN at FU (6.6 ± 3.4 months): 35% (n=10)  Full recovery rates BN at FU (6.6 ± 3.4 months): 28% (n=8) |
| Herzog, Field, Keller et al.  (1996) | n=229  (100%)  M_ED_=24.5 ± 6.7  (13- 45 years) | BN=153  AN=76 | Longitudinal  study  FU= 4 years | DSM-III-R | Full recovery ED: PSR ≤ 2 for 8 consecutive weeks.  Partial recovery ED: PSR ≤ 4 for 8 consecutive weeks. | Partial recovery rates BN at FU: 86.3% (n=132)  Full recovery rates BN at FU: 56.2% (n=86) |
| Herzog, Hopkins & Burns  (1993) | n=138  (100%)  M_ED_=26.7 ± 7.3 | SBN=15  SAN=8  SAN/SBN=  10 | Longitudinal  study  FU= 41 months | DSM-III-R | Recovery ED: 1) weighing at least 90% of ideal body weight and having regular menses for at least the past 3 months; 2) lacking fear of weight gain and the body image disturbance, showing no overconcern with body shape and weight; 3) binging and purging less than once a month for the past 3 months. | Recovery rates SBN at FU: 20% (n=3) |
| Herzog, Keller, Lavori et al.  (1988) | n=30  (100%)  M=23.9 ± n/a | BN | Longitudinal  study  FU=6 months | DSM–III | Recovery: PSR ≤ 2 for 8 consecutive weeks. | Recovery rates at FU: 33% (n=10) |
| Herzog, Sacks, Keller et al.  (1993) | n=229  (100%)  M_BN_= 22.8 ± 7.4 | BN=98  AN=41 AN/BN=90 | Longitudinal  study  FU=1 year | DSM-III-R | Full recovery ED: PSR ≤ 2 for 8 consecutive weeks.  Partial recovery ED: PSR ≤ 4 for at least 8 consecutive weeks; or PSR ≤ 2 for less than 8 consecutive weeks. | Partial recovery rates BN at FU: 71% (n=68)  Full recovery rates BN at FU: 56% (n=53) |
| Hsu & Sobkiewicz  (1989) | n=45  (100%)  M=n/a  (16-36 years) | BN | Longitudinal study  FU=4-6 years | DSM-III-R | Recovery: absence of bingeing/vomiting/ purging at all for the previous six months. | Recovery rates at FU: 60% (n=21) |
| Jacobi, Beintner, Fittig et al.  (2017) | n=253  (100%)  M=25.9 ± 7.1 | BN | RCT  Web-based aftercare program vs TAU  FU=18 months | DSM-IV-TR | Recovery: not fulfilling DSM-IV-TR diagnostic criteria for an eating disorder anymore at FU. | Recovery rates at PT: 40.1% (n=67/167)  Recovery rates at FU: 50.6% (n=83/164) |
| Keshishian, Tabri, Becker et al.,  (2019) | n=246  (100%)  M_ED_=46.3 ± 6.6 | BN=110  AN=136 | Longitudinal  study  FU=22 years | DSM-IV | Recovery ED: PSR ≤ 2 for 1 year. |  |
| Keski-Rahkonen, Hoek, Linna et al.  (2009) | n=211  (100%)  M=24.4 ± 0.9  (21–27 years) | BN=77  C=134 | Longitudinal  study  FU=5 years | DSM-IV | Clinical recovery: 1) BMI ≥19 kg/m^2^; 2) absence of bingeing and purging; for at least 1 year prior to assessment. | Recovery rates at FU: 55% (n=55). |
| Keski-Rahkonen, Raevuori, Bulik et al. (2012) | n=193  (100%)  M_BN_=25.5 ± 1.6  (21–27 years) | BN=59  C=134 | Cross-sectional study  BN vs C | DSM-IV | Clinical recovery: 1) BMI ≥19 kg/m^2^; 2) absence of bingeing and purging; for at least 1 year prior to assessment. |  |
| Klump, Strober, Bulik et al.,  (2004) | n=1238  (100%)  M_BN_= 27.1 ± 0.7  M_RBN_= 30.8 ± 1.2 | BN=279  AN=122  AN+BN=  267  EDNOS=63  HC=507 | Cross-sectional study  ED vs RED vs HC | DSM-IV | Recovery BN: binge/purge free for at least 1 year. |  |
| Kuipers, Hollander, Ark et al. (2017) | n=38  (100%)  M_ED_=22.2 ± 3.5 | BN=4  AN=27  EDNOS=7 | Longitudinal  study  FU= 18 months | DSM-IV-TR | Recovery ED: no longer fulfilling the criteria for an eating disorder diagnosis according to the SCID-I. | Recovery rates BN at FU: 75% (n=3) |
| Levallius, Roberts, Clinton et al.  (2016) | n=130  (100%)  M_ED_=28.3 ± 8.1 | BN=70  EDNOS=60 | Longitudinal  study  FU=6 months | DSM-IV | Recovery ED: not fulfilling any DSM-IV ED diagnosis over the last 90 days. | Recovery rates ED at FU: 70% (n=90) |
| Lock, Agras, Le Grange et al.  (2013) | n=358  (100%)  M_BN-adolescenti_=  15.8 ± 1.6  M_BN-adulti_=  30.8 ± 8.3 | BN_adolescenti_=49  BN_adulti_=101  BED_adulti_=97AN_adolescenti_=83  AN_Adulti_=28 | Retrospective study | DSM-IV | Recovery ED: 1) IBW >95% based on age, height, and gender; 2) Psychological-Global Score on the EDE less than community norms +1 SD; 3) no reported binges or compensatory behaviors as measured by EDE. |  |
| Melisse, Dekker, Van den Berg et al.  (2022) | n=625  (BN=97.3%)  M_BN_= 27.8 ± 7.7 | BN=370  BED=113  OSFED=142 | Longitudinal  study  FU= 20 weeks | DSM-5 | Recovery ED: 1) EDE-Q global score <2.77 and no additional eating disorder behaviours (binging, purging, laxatives, exercising) during the last month , 2) BMI ≥ 18,5 kg/m^2^. | Recovery rates BN at EOT: 31% (n=115)  Recovery rates BN at FU: 9.1% (n=34) |
| Mitchell, Agras, Crow et al.  (2011) | n=293  (n/a %)  M=29.6 ± 8.9 | BN | RCT  CBT+fluoxetina vs stepped-care  FU=1 year | DSM–IV | Recovery: no binge eating or purging behaviours for 28 days. | Recovery rates at EOT: 13.1% (n=31/237)  Recovery rates at FU: 21.8% (n=51/233) |
| Murray, Tabri, Thomas et al.  (2017) | n=225  (100%)  M_ED_=24.0 ± 6.6 | BN=106  AN=119 | Longitudinal  study  FU= 22 years | DSM-IV | Recovery ED: PSR ≤ 2 for 1 year. | Recovery rates BN at FU: 49% (n=52) |
| Nakai, Nin, Noma et al. (2014) | n=233  (100%)  M_BN_= 21.2 ± 4.0 | BN=85  AN=98  AN/BN=39  EDNOS=11 | Longitudinal  study  FU=7 years | DSM-IV | Full recovery ED: 1) BMI ≥ 18.0 kg/m^2^ and no abnormality in menstrual pattern; 2) no abnormality in eating behaviours (dietary restriction, binge eating and purging); 3) no abnormality in perception of body weight and shape (psychological state); for at least 3 consecutive months.  Partial recovery ED: reduction of symptoms to less than full criteria for at least 3 consecutive months. | Partial recovery rates BN at FU: 22.3% (n=19)  Full recovery rates BN at FU: 46% (n=39) |
| Reas, Williamson, Martin et al.  (2000) | n=44  (100%)  M=21.1 ± 5.6  (13–40 years) | BN | Longitudinal  study  FU=9 years | DSM-III | Recovery: having no scale elevations (t score≥ 70) on the binge eating, purging, or restrictive eating scales of the MAEDS. | Recovery rates at FU: 72.7% (n=32) |
| Rossotto, Rorty-Greenfield & Yager  (1996) | n=80  (100%)  M=24 ± n/a  (18-35 years) | BN | Cross-sectional study  recovered vs not-recovered | DSM-IV | Recovery: not experienced a clinical or subclinical ED, for a minimum of one year prior to the study. |  |
| Shaw, Herzog, Clark et al.,  (2012) | n=110  (100%)  M= 21.9 ± 5.6 | BN | Longitudinal  study  FU=8 years | DSM-IV | Recovery: PSR ≤ 2 for 8 consecutive weeks. | Recovery rates at FU: 79.1% (n=87) |
| Silén, Sipilä, Raevuori et al.  (2021) | n=145  (89.4%)  M_ED_=22.4 ± n/a | BN=18  BED=6  AN=46  OSFED=33  UFED=42 | Retrospective study | DSM‐5 | Recovery ED: 1) no ED behaviour (e.g., restrictive eating or binge eating, compensatory behaviours, excessive exercise), 2) no psychological symptoms (e.g., persistent body image concerns, fear of weight gain, fatphobia) during the last year, 3) BMI≥18,5 kg/m^2^. | Recovery rates BN at FU (5 years): 23.1% (n=4) |
| Stein, Kaye, Matsunaga et al.,  (2002) | n=25  (100%)  M_RBN_=28.8 ± 5.5 | RBN=11  HC=15 | Cross-sectional study  RBN vs HC | DSM-IV | Recovery: 1) normal weight and regular menstrual cycles; 2) absence of binging, purging or restricting behaviors; for at least 1 year. |  |
| Von Holle, Pinheiro, Thornton et al.  (2008) | n=901  (100%)  M_ED_=28.0 ± 8.1  (15-58 years) | BN=276  AN=625 | Retrospective study | DSM–IV | Recovery ED: 1) any eating disorders symptoms (i.e. low weight, dieting, binge-eating, compensatory behaviours), at least for 3 years. Subthreshold psychological features such as weight influencing self-evaluation were allowed based on the fact that low levels ofthese features represent ‘normative discontent. | Recovery rates BN at FU (15 years): 25% (n=69)  Recovery rates BN at FU (20 years): 19% (n=52) |
| von Ranson, Kaye, Weltzin et al.,  (1999) | n=79  (100%)  M_BN_= 19.8 ± 5.3  M_RBN_=25.4 ± 5.3 | BN=31  RBN=29  HC=19 | Cross-sectional study  BN vs RBN vs HC | DSM-III-R | Recovery: 1) normal weight and regular menstrual cycles; 2) absence of binging, purging or restricting behaviors; for at least 1 year. |  |
| Wagner, Barbarich-Marsteller, Frank et al.  (2006) | n=107  (100%)  M_RBN_=26.0 ± 5.8  (18-45 years) | RBN=19  RAN=41  C=47 | Cross-sectional study  RBN vs RAN vs C | DSM-IV | Recovery ED: 1) maintain a weight > 90% of average body weight and have regular menstrual cycles; 2) have not binged, purged, restricted food intake, or exercised excessively; 3) not used psychoactive medications such as antidepressants; 4) not have met criteria for current alcohol or drug abuse/ dependence; criteria met for 1 year. |  |
| **Articles giving definitions of relapse** | | | | | | |
| Bergh, Brodin, Lindberg et al.  (2002) | n=168  (97%)  M_BN_=19 ± n/a  (15–54 years) | BN=38  AN=85  EDNOS=45 | RCT  treatment VS no treatment  FU= 60 months | DSM-IV | Relapse ED: patient fulfilled the criteria of an ED. | Relapse rates of ED at FU (1 year): 7% (n=6) |
| Bergh, Brodin, Maletz et al.  (2013) | n=1.428  (97%)  M_BN_=22.6 ± n/a | BN= 246  AN=571  EDNOS= 611 | Longitudinal  study  FU=60 months | DSM-IV | Relapse ED: patient fulfilled the criteria of an ED. | Relapse rates of ED at FU (2 years): 10% (n=74) |
| Bohon, Stice & Burton (2009) | n=96  (100%)  M=19.7 ± 4.8 | BN=42  S-BN=54 | Longitudinal  study  FU=1 year | DSM-IV | Relapse: engaging in 2 or more episodes of binge eating or compensatory behavior after a period of remission. |  |
| Commerford, Licinio & Halmi  (1997) | n=31  (96.7%)  M= 19.8 ± 1.6  (14-52 years) | BN=9  AN=19  AN+BN=3 | Longitudinal  study  FU=5 years | DSM-III-R | Relapse ED: currently meeting diagnostic criteria for ED. | Relapse rates of ED at FU: 48% (n=15) |
| Fairburn, Cooper, Doll et al.  (2000) | n=150  (100%)  M_BN_=23.9 ± 5.0  (16-35 years) | BN=102  BED=48 | Longitudinal  study  FU=5 years | DSM-IV | Relapse ED: having any DSM-IV ED. | Relapse rates of BN at FU (3 years): 32%(n=8/25)  Relapse rates of BN at FU (4 years): 33%(n=9/27)  Relapse rates of BN at FU (5 years): 26%(n=8/31) |
| Fairburn, Peveler, Jones et al.,  (1993) | n=75  (100%)  M=n/a | BN | RCT  CBT vs BT vs IPT  FU=1 year | DSM-III-R | Relapse: return of binge eating and compensatory behaviour (vomiting and use of laxatives). | Relapse rates at FU: 16% (n=12) |
| Grilo, Pagano, Stout et al. (2012) | n=117  (100%)  M_ED_=31.2 ± 8.1 (18-45 years) | BN= 35 EDNOS=82 | Longitudinal  study  FU= 6 years | DSM-IV | Relapse ED: PSR≥ 2 for 8 consecutive weeks. | Relapse rates of BN at FU: 46% (n=16) |
| Keel, Dorer, Franko et al.  (2005) | n=246  (100%)  M_BN_=24.3 ± n/a | BN=110  AN=136 | Longitudinal  study  FU=9 years | DSM-IV | Relapse ED: PSR=5 or 6 (return to full syndromal criteria after a period of remission). | Relapse rates of BN at FU: 35% (n=38) |
| MacDonald,  Trottier, McFarlane et al.  (2015) | n=158  (96.2%)  M_ED_=27.1 ± 8.8  (17-57 years) | BN=139  OSFED-PD=  19 | Longitudinal  study  FU=12 months | DSM-5 | Relapse ED: average of 4 or more binge and/or vomit episodes per month, for 3 consecutive months. | Relapse rates of BN at FU (6 months): 37.6% (n=38)  Relapse rates of BN at FU (12 months): 51.1% (n=46) |
| Mcfarlane,  Olmsted & Trottier (2008) | N=58  (100%)  M_BN_= 30.7 ± 11.8 | BN=18  AN=16  EDNOS=24 | Longitudinal  study  FU=24 months | DSM-IV | Relapse BN: more than 8 binge eating and/or purging episodes per month, for 3 consecutive months. | Relapse rates of BN at FU: 28% (n=5) |
| Mitchell, Davis & Goff (1985) | n=30  (n/a)  M=24.8 ± n/a  (18-40 years) | BN | Longitudinal  study  FU=12-15 months | DSM-III | Relapse: reoccurrence of a regular pattern of bulimic behaviors, including binge-eating and either self-induced vomiting or abusing laxatives, at a minimum frequency of once each week, for at least 2 months. | Relapse rates at FU (1-3 months): 40% (n=12) |
| Olmsted, Kaplan & Rockert (1994) | n=48  (100%)  M=25.8 ± 7.1  (18 - 57 years) | BN | Longitudinal  study  FU=2 years | DSM-III-R | Relapse: mean of 2 episodes of binge and/or vomiting for week, for at least 3 months. | Relapse rates at FU: 31.3% (n=15) |
| Olmsted, Kaplan & Rockert (2005) | n=54  (100%)  M=26.5 ± 6.1 | BN | Longitudinal  study  FU=19 months | DSM-III-R | Relapse: mean of 2 episodes of binge and/or vomiting for week, for at least 3 months. | FU relapse rates varied according to the definitions of remission and relapse used. The lowest relapse rate (21%, n=7) occurred in patients who were abstinent at baseline and relapse was defined as the presence of at least two symptoms per week for 3 months. The highest relapse rate (55%, n=25) occurred in patients who were abstinent at baseline (up to 3 episodes) and relapse was defined as the presence of at least two symptoms per week for at least 1 month. |
| Olmsted, Kaplan, Rockert et al. (1996) | n=166  (100%)  M=25.3 ± 6.6 | BN | Longitudinal  study  FU=2 years | DSM-III-R | Relapse: mean of 2 episodes of binge and/or vomiting for week, for at least 3 months. | Relapse rates at FU: 24% (n=40) |
| Olmsted, McFarlane,  MacDonald et al.  (2015) | n=116  (100%)  M= 27.2 ± 9.5 | BN  [n_rapid relapse_=32;  n_no rapid relapse_=84] | Longitudinal  study  FU=12 months | DSM-IV | Relapse: mean of 8 episodes (binge and/or vomit) per month, for 3 consecutive months. | Relapse rates of BN at FU (6 months): 27.6% (n=32) |
| Pyle, Mitchell, Eckert et al. (1990) | n=68  (100%)  M=n/a  (18-40 years) | BN | Longitudinal  study  FU=6 months | DSM-III | Relapse: binge-eating coupled with self-induced vomiting or laxative abuse on 8 or more occasions in a 4-week period. | Relapse rates at FU: 30% (n=18) |
| Romano, Halmi, Sarkar et al.  (2002) | n=150  (98%)  M= 29.7 ± 8.2 | BN  [n_fluoxetine_=76, n_placebo_=74] | RCT  fluoxetine vs P  FU=52 weeks | DSM-IV | Relapse: return to the baseline frequency of vomiting for 2 consecutive weeks. | Relapse rates of BN at FU (3 months): 39.3% (n=59) |
| Sollid, Clausen, Maimburg  (2021) | n=122  (100%)  M_ED_=n/a | BN=28  BED=4  AN=84  s-ED=6 | Longitudinal  study  FU=6-8 post-partum weeks | ICD-10 | Relapse ED: onset of recurrent ED behavior at least once a week over a period of a month. | Relapse rates of BN: 28.6% (n=8/28) |
| Walsh, Hadigan, Devlin et al.  (1991) | n=78  (100%)  M=24.8 ± 4.5  (18-45 years) | BN | RCT  desipramine vs P  FU=6 months | DSM-III-R | Relapse: binge at more than 50% of their baseline binge frequency for 2 consecutive weeks. | Relapse rates at FU: 29% (n=6) |
| **Articles giving definitions of both recovery and relapse** | | | | | | |
| Castellini, Lo Sauro, Mannucci et al.  (2011) | n=793  (BN=97.1%)  M_BN_= 26.6 ± 7.8  (18-60 years) | BN=137, BED=262, AN=165, EDNOS=  229 | Longitudinal  study  FU=6 years | DSM-IV | Recovery ED: not fulfill the DSM-IV criteria for any ED (including EDNOS).  Relapse ED: return to a full syndromal or EDNOS criteria after a period of remission. | Recovery rates for BN at FU: 49.6% (n=68)  Relapse rates for BN: 17.7% (n=12) |
| Clausen (2008) | n=78  (n/a)  M_BN_= 22.2 ± 3.5 | BN=30  AN=35  EDNOS=13 | Longitudinal  study  FU=2.5 years | DSM-IV | Recovery ED: PSR=1  Relapse ED: fulfilling all criteria of an ED after having been in remission, for at least 3 months | Recovery/remission rates BN at FU: 36.7% |
| Field, Herzog, Keller et al. (1997) | n=106  (100%)  M=n/a | BN | Longitudinal  study  FU=36 months | DSM-III-R | Recovery: abstinence from binge eating and compensatory behaviour for at least 1 year (less likelihood of relapse).  Relapse: 1) binge and compensatory behaviour at least once a week, for 4 weeks; or 2) binge (with or without compensatory behaviour) at least twice a week, for 4 weeks. | Recovery rates at FU (1 year): 37.7% (n=40)  Relapse rate (at 3 months): 25% (n=26) |
| Herzog, Dorer, Keel et al.  (1999) | n=246  (100%)  M_BN_=25.5 ± 6.5  (13-45 years) | BN=110  AN=136 | Longitudinal  study  FU=7.5 years | DSM-IV | Full recovery ED: PSR ≤ 2 for at least 8 consecutive weeks.  Partial recovery ED: PSR ≤ 4 for at least 8 consecutive weeks.  Relapse: PSR= 5 o 6 for at least 8 consecutive weeks after being in full recovery. | Partial recovery rates for BN at FU (2 years): 88% (n=97)  Full recovery rates for BN at FU (2 years): 53% (n=58)  Partial recovery rates for BN at FU (7 years): 98% (n=108)  Full recovery rates for BN at FU (7 years): 73% (n=80)  Relapse rate for BN: 35.3% (n=39) |
| Keller, Herzog, Lavori et al. (1992) | n=30  (100%)  M=23.9 ± n/a | BN | Longitudinal  study  FU=35-42 months | DSM-III | Recovery: PSR ≤ 2 for at least 8 consecutive weeks.  Relapse: meeting full DSM-III criteria for BN, for a minimum of 2 consecutive weeks. | Recovery rates at FU: 69% (n=21)  Relapse rate (after 78 weeks): 63% (n=9) |
| Kordy, Palmer, Papezova et al.  (2002) | n=655  (BN=97.7%)  M_BN_=25.9 ± 6.3 | BN=422  AN=233 | Longitudinal  study  FU=2.5 years | DSM-III-R | Recovery BN: no binge or compensatory behaviours, no weight reduction with vomiting or laxative abuse, for at least 12 months.  Relapse BN: change from partial or complete remission to a complete syndrome according to DSM-IV. | Recovery rates BN at FU: 16% (n=68) |
| Larrañaga,  Fluiters, Docet et al. (2014) | n=77  (BN=100%)  M_BN_= 22.0 ± 3.6  (15-53 years) | BN=19  AN=32  EDNOS=23 | Longitudinal  study  FU=3 years | DSM-IV-TR | Recovery ED: 1) psychological symptom-free (for EAT-26 and QEWP-R), 2) nutritional symptom-free (muscle weakness, depression, anemia, fatigue, mouth lesions, edema, low blood pressure, etc.), 3) a normal lifestyle according to the patients’ family, social and professional conditions; at least for 12 months after the treatment.  Relapse ED: returning to full symptomatic status after being in full recovery. | Recovery rates BN: 42.1% (n=8) |
| Richard, Bauer, Kordy et al.  (2005) | n=1171  (BN=97.7%)  M_BN_=25.9±6.3 | BN=647  AN=355  AN/BN=  169 | Longitudinal  study  FU=2.5 years | DSM-III-R | Recovery BN: no binge or compensatory behaviours, no weight reduction with vomiting or laxative abuse, for at least 12 months.  Relapse BN: change from partial or complete remission to a complete syndrome according to DSM-IV. | Recovery/remission rates at FU: 74% (n=313)  Relapse rates BN: 37.4% (n=158) |
| Stice, Marti, Shaw et al. (2009) | n=496  (100%)  M_ED_= 13 ± n/a  (12-15 years) | BN=38  BED=28  AN=6  PD=22 | Longitudinal  study  FU=8 years | DSM-IV | Recovery ED: not satisfying criteria of DSM-IV for a particular ED, for at least a 1-month period.  Relapse ED: meeting criteria for another episode of an ED (as defined in DSM-IV), after showing recovery from the same ED. | Recovery rates for BN at FU: 91% (n=29)  Relapse rates BN: 41% (n=13) |
| Yu, Agras & Bryson (2013) | n=96  (n/a)  M= 30.9 ± 8.4 | BN | Longitudinal  study  FU=62 weeks | DSM-IV | Recovery: 1a) diagnostic criteria: less than 8 episodes of binge eating and compensatory behaviour, or 1b) behavioural criteria: abstinence from binge eating and compensatory behaviour in the last 4 weeks 2) BMI ≥ 18.5 kg/m2; 3) EDE Global Score within 1 SD in the normal range.  Relapse: not fulfilling the criteria for recovery at the FU after meeting the criteria for recovery at the EOT. | Using the behavioural criterion of absence of diagnosis:  Recovery rates at EOT: 51% (n=49)  Recovery rates at FU: 40.6% (n=39)  Relapse rate: 32.7% (n=16)  Using abstinence from pathological behaviour as a criterion:  Recovery rates at EOT: 15.6% (n=15)  Recovery rates at FU: 20.8% (n=20)  Relapse rate: 60% (n=9)  With the full definition of recovery (criteria 1+2+3) and the diagnostic behavioural criterion (1a):  Recovery rates at FU: 28.1% (n=27)  Relapse rate: 14.6% (n=14)  Using the full definition of recovery and the behavioural criterion of abstinence (1 b):  Recovery rates at FU: 16.7% (n=16)  Relapse rate: 8.3% (n=8) |

**Abbreviations:** AN=Anorexia Nervosa; RAN= Recovered AN; BN=Bulimia Nervosa; RBN=Recovered BN; s-BN= subthreshold BN; BED=Binge Eating Disorder; s-BED= subthreshold BED; EDNOS=Eating Disorder Not Otherwise Specified; PD=Purging Disorder; OSFED=Other Specified Feeding and Eating Disorder; UFED=Unspecified Feeding or Eating Disorders; ED=Eating Disorder; AED=Active ED; RED=Recovered ED;

DSM=Diagnostic and Statistical Manual of Mental Disorders; ICD= International Classification of Diseases; EDE= Eating Disorder Examination; EDE-Q=Eating Disorders Examination Questionnaire; EAT-26= Eating Attitude Test-26 Item; PSR= Psychiatric Status Rating; QEWP-R=Questionnaire of Eating and Weight Patterns Revised; SCID= Structured Clinical Interview; MAEDS=Multiaxial Assessment of Eating Disorders Symptoms; FR=Fully Recovered; PR=Partially Recovered; IBW=Ideal Body Weight, BMI=body mass index. EOT=End Of Treatment; PT=Post-Treatment; FU=Follow-Up;

RCT=Randomized Controlled Trial; CBT= Cognitive Behavioral Therapy; IPT= Interpersonal Psychotherapy TAU= Treatment As Usual; P=placebo; C=control group; HC=Healty Control; SD=Standard Deviation; n/a=not available.

| CRITERIA | STRONG | MODERATE | WEAK |
| --- | --- | --- | --- |
| Research Design | Randomized Control Trial or experimental study | Observational cohort or case-control  studies | Uncontrolled studies |
| Was the research objective clearly stated and directly related to review topic? | Clear description of objective. Outcome measures directly related to topic | Moderately clear, some details missing.  Some outcome measures related to topic | Unclear or not stated |
| Was the study population clearly defined  with inclusion/exclusion criteria stated and consistent? | Clear description of population and  inclusion/exclusion criteria | Moderately clear, some details missing | Unclear or not stated |
| Comparator: were the subjects selected  from a comparable population in all  respects? (Diagnosis, age, gender, weight, BMI, severity of disease, previous  hospitalizations, duration of illness) | Participants were comparable in at least:  Diagnosis, age, weight, BMI, severity of  disease, timeframe of treatment, similar  duration of illness, and number of previous hospitalizations | Participants were comparable in at least three between:  Age, sex, schooling, ethnicity, marital status, BMI, diagnosis. Some differences reported in timeframe of treatment, severity of disease, duration of illness, or number of previous hospitalizations. | No comparator group or unclear or not  stated |
| Sample size | >100 | 50-99 | <50 |
| Was the follow-up timeframe sufficient? | 1 year or greater follow-up | 3 months to 11 months follow-up | < 3 months follow-up |
| Was treatment thoroughly described? | Clear description of regimen and  formula | Moderately clear, some details missing | Unclear or not stated |
| For outcomes that can vary, did the study  clearly define different levels of the  outcome? (number and severity of binge/purge episodes) | Clear description, different levels of  outcomes reported | Moderately clear description of the different levels of outcomes | No alternate level of outcome reported |
| Data Collection Method: scale, methods to collect binge/purge data | Tools are valid and reliable | Tools are valid but reliability not described | No evidence of validity or reliability or not  stated |
| Measurement Bias: Were the outcome  measures clearly defined, valid, reliable  and implemented consistently? | Valid, reliable, and explained in detail | Measurement valid but reliability not described | Self-reported by participants or not stated  or unclear |
| Selection Bias: Is study sample  representative of target population and if <100% eligible cases were selected, were  they randomized? | Very likely to be representative of target population, >80% participation rate | Somewhat likely to be representative of target population, 60-79% participation | < 60% participation rate or not stated |
| Attrition Bias: Was loss to follow-up after  baseline minimized? | >80% follow-up after baseline | 60-79% follow-up after baseline and explanation of those lost | < 60% follow-up after baseline or not reported |
| Confounders: Were key potential  confounding variables measured and  adjusted statistically for their impact on  the relationship between treatment and  outcome? | Confounders identified, discussed, and  adjusted for statistically | Confounders identified and discussed | Unclear or not stated |

**Supplementary Table S2.** *Quality assessment and risk of bias criteria for observational cohort, case-control, and controlled intervention studies*

Format modified from National Institutes of Health (2021)

**Supplementary Table S3.** *Assessment of quality and risk of bias of selected experimental studies*

| CRITERIA | *Bailer et al., 2004* | *Bardone-Cone et al., 2016* | *Bloks et al., 2004* | *Brewerton et al., 2011* | *Cabelguen et al., 2023* | *Castellini et al., 2014* | *Castellini et al., 2017* | *Castellini et al., 2012* |
| --- | --- | --- | --- | --- | --- | --- | --- | --- |
| Research Design | S | M | M | M | W | M | M | M |
| Was the research objective clearly stated and directly related to review topic? | M | S | S | M | M | W | M | M |
| Was the study population clearly defined with inclusion/exclusion criteria stated and consistent? | M | M | M | M | M | M | S | S |
| Comparator: were the subjects selected from a comparable population in all respects? | S | M | W | M | W | W | W | M |
| Sample size | M | S | S | S | S | S | M | S |
| Was the follow-up timeframe sufficient? | S | N/A | S | S | S | S | S | S |
| Was treatment thoroughly described? | S | N/A | N/A | W | N/A | N/A | N/A | M |
| For outcomes that can vary, did the study clearly define different levels of the outcome? | S | S | S | S | W | M | M | M |
| Data Collection Method: scale, methods to collect data | M | S | S | M | M | M | M | M |
| Measurement Bias: Were the outcome measures clearly defined, valid, reliable and implemented consistently? | M | S | M | W | W | W | W | M |
| Selection Bias: Is study sample representative of target population and if <100% eligible cases were selected, were they randomized? | W | M | M | W | S | S | M | S |
| Attrition Bias: Was loss to follow-up after baseline minimized? | M | N/A | S | S | W | S | W | S |
| Confounders: Were key potential confounding variables measured and adjusted statistically for their impact on the relationship between treatment and outcome? | S | N/A | N/A | M | N/A | N/A | N/A | M |
| Total Points (S=3; M=2; W=1; N/A=0) | 31 | 23 | 27 | 27 | 20 | 23 | 21 | 31 |
| Overall Score (0-25 = W; 26-30 = M; >31= S) | **S** | **W** | **M** | **M** | **W** | **W** | **W** | **S** |

| CRITERIA | *Cogley et al., 2003* | *De Young et al., 2020* | *Eddy et al., 2008* | *Eddy et al., 2007* | *Eddy et al., 2017* | *Eielsen et al., 2021* | *Forney et al., 2022* | *Franko et al., 2008* |
| --- | --- | --- | --- | --- | --- | --- | --- | --- |
| Research Design | M | M | M | M | M | M | M | M |
| Was the research objective clearly stated and directly related to review topic? | S | S | M | M | S | M | M | M |
| Was the study population clearly defined with inclusion/exclusion criteria stated and consistent? | S | M | M | M | M | S | M | S |
| Comparator: were the subjects selected from a comparable population in all respects? | M | S | M | M | M | M | S | S |
| Sample size | S | S | S | S | S | M | S | S |
| Was the follow-up timeframe sufficient? | N/A | S | S | S | S | S | S | S |
| Was treatment thoroughly described? | N/A | N/A | N/A | N/A | N/A | N/A | N/A | N/A |
| For outcomes that can vary, did the study clearly define different levels of the outcome? | S | S | S | S | S | S | S | S |
| Data Collection Method: scale, methods to collect data | S | M | M | M | S | S | S | S |
| Measurement Bias: Were the outcome measures clearly defined, valid, reliable and implemented consistently? | M | S | S | S | S | S | S | S |
| Selection Bias: Is study sample representative of target population and if <100% eligible cases were selected, were they randomized? | S | M | S | S | M | M | S | S |
| Attrition Bias: Was loss to follow-up after baseline minimized? | N/A | S | S | S | M | M | S | S |
| Confounders: Were key potential confounding variables measured and adjusted statistically for their impact on the relationship between treatment and outcome? | N/A | N/A | N/A | N/A | N/A | N/A | N/A | N/A |
| Total Points (S=3; M=2; W=1; N/A=0) | 24 | 29 | 28 | 28 | 28 | 27 | 30 | 31 |
| Overall Score (0-25 = W; 26-30 = M; >31= S) | **W** | **M** | **M** | **M** | **M** | **M** | **M** | **S** |

| CRITERIA | *Franko et al.,*  *2005* | *Franko et al.,*  *2018* | *Garte et al.,*  *2015* | *Harrison et al., 2014* | *Harrison et al.,2011* | *Hergenroeder, et al., 2015* | *Herzog et al.,*  *1996* | *Herzog, Hopkins et al.,*  *1993* |
| --- | --- | --- | --- | --- | --- | --- | --- | --- |
| Research Design | M | M | M | M | M | W | M | W |
| Was the research objective clearly stated and directly related to review topic? | M | S | M | S | M | M | M | W |
| Was the study population clearly defined with inclusion/exclusion criteria stated and consistent? | S | M | M | M | S | M | M | M |
| Comparator: were the subjects selected from a comparable population in all respects? | M | S | M | S | S | M | M | W |
| Sample size | S | S | M | S | S | S | S | W |
| Was the follow-up timeframe sufficient? | S | S | W | N/A | N/A | N/A | S | S |
| Was treatment thoroughly described? | N/A | N/A | S | N7A | N/A | N/A | N/A | N/A |
| For outcomes that can vary, did the study clearly define different levels of the outcome? | S | S | M | S | S | S | S | M |
| Data Collection Method: scale, methods to collect data | M | M | M | S | S | W | M | W |
| Measurement Bias: Were the outcome measures clearly defined, valid, reliable and implemented consistently? | S | S | M | M | W | W | S | W |
| Selection Bias: Is study sample representative of target population and if <100% eligible cases were selected, were they randomized? | S | S | S | S | M | M | W | M |
| Attrition Bias: Was loss to follow-up after baseline minimized? | W | M | M | N/A | N/A | N/A | S | S |
| Confounders: Were key potential confounding variables measured and adjusted statistically for their impact on the relationship between treatment and outcome? | N/A | N/A | W | N/A | N/A | N/A | N/A | N/A |
| Total Points (S=3; M=2; W=1; N/A=0) | 27 | 29 | 26 | 24 | 22 | 17 | 26 | 18 |
| Overall Score (0-25 = W; 26-30 = M; >31= S) | **M** | **M** | **M** | **W** | **W** | **W** | **M** | **W** |

| CRITERIA | *Herzog et al.,*  *1988* | *Herzog, Sacks et al., 1993* | *Hsu et al.,*  *1989* | *Jacobi et al.,*  *2017* | *Keshishian*  *et al., 2019* | *Keski-Rahkonen*  *et al., 2009* | *Keski-Rahkonen*  *et al., 2012* | *Klump et al.,*  *2004* |
| --- | --- | --- | --- | --- | --- | --- | --- | --- |
| Research Design | W | M | W | S | M | M | M | M |
| Was the research objective clearly stated and directly related to review topic? | M | M | M | M | M | M | M | M |
| Was the study population clearly defined with inclusion/exclusion criteria stated and consistent? | M | M | M | S | M | M | M | S |
| Comparator: were the subjects selected from a comparable population in all respects? | W | S | W | S | M | W | S | S |
| Sample size | W | S | W | S | S | S | S | S |
| Was the follow-up timeframe sufficient? | M | S | S | S | S | S | N/A | N/A |
| Was treatment thoroughly described? | N/A | N/A | N/A | S | N/A | N/A | N/A | N/A |
| For outcomes that can vary, did the study clearly define different levels of the outcome? | S | S | M | M | S | S | S | M |
| Data Collection Method: scale, methods to collect data | M | M | W | M | M | S | S | M |
| Measurement Bias: Were the outcome measures clearly defined, valid, reliable and implemented consistently? | S | S | W | M | S | M | M | W |
| Selection Bias: Is study sample representative of target population and if <100% eligible cases were selected, were they randomized? | M | W | M | W | S | M | S | S |
| Attrition Bias: Was loss to follow-up after baseline minimized? | S | S | M | M | M | S | N/A | N/A |
| Confounders: Were key potential confounding variables measured and adjusted statistically for their impact on the relationship between treatment and outcome? | N/A | N/A | N/A | S | N/A | N/A | N/A | N/A |
| Total Points (S=3; M=2; W=1; N/A=0) | 22 | 27 | 18 | 32 | 27 | 26 | 23 | 21 |
| Overall Score (0-25 = W; 26-30 = M; >31= S) | **W** | **M** | **W** | **S** | **M** | **M** | **W** | **W** |

| CRITERIA | *Kuipers et al., 2017* | *Levallius et al., 2016* | *Lock et al.*  *2013* | *Melisse et al.,*  *2022* | *Mitchell et al., 2011* | *Murray et al.,*  *2017* | *Nakai et al., 2014* | *Reas et al., 2000* |
| --- | --- | --- | --- | --- | --- | --- | --- | --- |
| Research Design | M | M | M | M | S | M | M | M |
| Was the research objective clearly stated and directly related to review topic? | M | M | S | M | M | M | S | M |
| Was the study population clearly defined with inclusion/exclusion criteria stated and consistent? | M | M | W | M | M | S | M | S |
| Comparator: were the subjects selected from a comparable population in all respects? | S | M | M | S | S | M | S | M |
| Sample size | W | S | S | S | S | S | S | W |
| Was the follow-up timeframe sufficient? | S | M | N/A | M | S | S | S | S |
| Was treatment thoroughly described? | M | S | N/A | M | S | N/A | M | M |
| For outcomes that can vary, did the study clearly define different levels of the outcome? | M | S | S | S | M | S | S | M |
| Data Collection Method: scale, methods to collect data | S | S | M | S | M | M | S | S |
| Measurement Bias: Were the outcome measures clearly defined, valid, reliable and implemented consistently? | W | M | M | M | M | W | M | M |
| Selection Bias: Is study sample representative of target population and if <100% eligible cases were selected, were they randomized? | M | S | S | M | M | S | M | W |
| Attrition Bias: Was loss to follow-up after baseline minimized? | S | M | N/A | W | M | M | S | S |
| Confounders: Were key potential confounding variables measured and adjusted statistically for their impact on the relationship between treatment and outcome? | M | S | N/A | M | S | N/A | W | M |
| Total Points (S=3; M=2; W=1; N/A=0) | 28 | 32 | 21 | 29 | 32 | 26 | 32 | 28 |
| Overall Score (0-25 = W; 26-30 = M; >31= S) | **M** | **S** | **W** | **M** | **S** | **M** | **S** | **M** |

| CRITERIA | *Rossotto et al.,*  *1996* | *Shaw et al.,*  *2012* | *Silén et al.,*  *2021* | *Stein et al.,*  *2002* | *Von Holle et al., 2008* | *von Ranson et al., 1999* | *Wagner et al.,*  *2006* | *Bergh et al.,*  *2002* |
| --- | --- | --- | --- | --- | --- | --- | --- | --- |
| Research Design | M | M | M | M | M | M | M | S |
| Was the research objective clearly stated and directly related to review topic? | M | M | M | S | M | M | S | S |
| Was the study population clearly defined with inclusion/exclusion criteria stated and consistent? | W | M | M | M | S | M | M | M |
| Comparator: were the subjects selected from a comparable population in all respects? | S | W | S | S | M | M | S | M |
| Sample size | M | S | S | W | S | M | S | S |
| Was the follow-up timeframe sufficient? | N/A | S | N/A | N/A | N/A | N/A | N/A | S |
| Was treatment thoroughly described? | N/A | N/A | N/A | N/A | N/A | N/A | N/A | S |
| For outcomes that can vary, did the study clearly define different levels of the outcome? | M | S | M | M | S | S | M | M |
| Data Collection Method: scale, methods to collect data | M | M | M | M | M | W | S | M |
| Measurement Bias: Were the outcome measures clearly defined, valid, reliable and implemented consistently? | W | S | W | W | M | W | M | M |
| Selection Bias: Is study sample representative of target population and if <100% eligible cases were selected, were they randomized? | M | M | S | S | S | M | S | W |
| Attrition Bias: Was loss to follow-up after baseline minimized? | N/A | S | N/A | N/A | N/A | N/A | N/A | M |
| Confounders: Were key potential confounding variables measured and adjusted statistically for their impact on the relationship between treatment and outcome? | N/A | N/A | N/A | N/A | N/A | N/A | N/A | M |
| Total Points (S=3; M=2; W=1; N/A=0) | 17 | 26 | 20 | 19 | 22 | 17 | 23 | 30 |
| Overall Score (0-25 = W; 26-30 = M; >31= S) | **W** | **M** | **W** | **W** | **W** | **W** | **W** | **S** |

| CRITERIA | *Bergh et al.,*  *2013* | *Bohon et al., 2009* | *Commerford et al., 1997* | *Fairburn et al.*  *2000* | *Fairburn et al., 1993* | *Grilo et al. 2012* | *Keel et al.,*  *2005* | *MacDonald*  *et al., 2015* |
| --- | --- | --- | --- | --- | --- | --- | --- | --- |
| Research Design | M | M | W | M | S | M | M | M |
| Was the research objective clearly stated and directly related to review topic? | M | M | M | S | S | S | S | M |
| Was the study population clearly defined with inclusion/exclusion criteria stated and consistent? | M | M | M | M | M | S | M | M |
| Comparator: were the subjects selected from a comparable population in all respects? | M | S | M | M | S | M | M | M |
| Sample size | S | M | W | S | M | S | S | S |
| Was the follow-up timeframe sufficient? | S | S | S | S | S | S | S | S |
| Was treatment thoroughly described? | M | N/A | N/A | N/A | M | N/A | N/A | N/A |
| For outcomes that can vary, did the study clearly define different levels of the outcome? | M | M | M | M | M | S | S | S |
| Data Collection Method: scale, methods to collect data | M | S | W | M | M | S | M | S |
| Measurement Bias: Were the outcome measures clearly defined, valid, reliable and implemented consistently? | W | M | W | M | M | S | S | M |
| Selection Bias: Is study sample representative of target population and if <100% eligible cases were selected, were they randomized? | W | S | W | M | S | S | S | S |
| Attrition Bias: Was loss to follow-up after baseline minimized? | M | S | W | S | M | S | S | M |
| Confounders: Were key potential confounding variables measured and adjusted statistically for their impact on the relationship between treatment and outcome? | M | N/A | N/A | N/A | M | N/A | N/A | N/A |
| Total Points (S=3; M=2; W=1; N/A=0) | 26 | 27 | 17 | 26 | 31 | 31 | 29 | 27 |
| Overall Score (0-25 = W; 26-30 = M; >31= S) | **M** | **M** | **W** | **M** | **S** | **S** | **M** | **M** |

| CRITERIA | *Mcfarlane et al., 2008* | *Mitchell et al., 1985* | *Olmsted et al., 1994* | *Olmsted et al., 2005* | *Olmsted et al., 1996* | *Olmsted et al.,*  *2015* | *Pyle et al. 1990* | *Romano et al.*  *2002* |
| --- | --- | --- | --- | --- | --- | --- | --- | --- |
| Research Design | M | W | M | M | M | M | W | S |
| Was the research objective clearly stated and directly related to review topic? | S | M | S | S | M | S | M | M |
| Was the study population clearly defined with inclusion/exclusion criteria stated and consistent? | M | M | M | M | W | M | M | S |
| Comparator: were the subjects selected from a comparable population in all respects? | M | W | M | W | W | M | W | S |
| Sample size | M | W | W | M | S | S | M | S |
| Was the follow-up timeframe sufficient? | S | S | S | S | S | S | M | S |
| Was treatment thoroughly described? | N/A | N/A | M | N/A | M | N/A | M | M |
| For outcomes that can vary, did the study clearly define different levels of the outcome? | S | M | M | M | M | M | M | M |
| Data Collection Method: scale, methods to collect data | M | W | M | M | M | M | M | M |
| Measurement Bias: Were the outcome measures clearly defined, valid, reliable and implemented consistently? | M | M | M | M | W | S | W | W |
| Selection Bias: Is study sample representative of target population and if <100% eligible cases were selected, were they randomized? | W | M | M | M | S | S | S | M |
| Attrition Bias: Was loss to follow-up after baseline minimized? | S | S | M | S | S | M | M | M |
| Confounders: Were key potential confounding variables measured and adjusted statistically for their impact on the relationship between treatment and outcome? | N/A | N/A | M | N/A | W | N/A | W | S |
| Total Points (S=3; M=2; W=1; N/A=0) | 25 | 20 | 27 | 24 | 26 | 27 | 23 | 31 |
| Overall Score (0-25 = W; 26-30 = M; >31= S) | **W** | **W** | **M** | **W** | **M** | **M** | **W** | **S** |

| CRITERIA | *Sollid et al.,*  *2021* | *Walsh et al.*  *1991* | *Castellini*  *et al., 2011* | *Clausen (2008)* | *Field et al., 1997* | *Herzog et al.,*  *1999* | *Keller et al., 1992* | *Kordy et al.,*  *2002* |
| --- | --- | --- | --- | --- | --- | --- | --- | --- |
| Research Design | M | S | M | M | M | M | M | M |
| Was the research objective clearly stated and directly related to review topic? | M | M | S | S | S | S | M | S |
| Was the study population clearly defined with inclusion/exclusion criteria stated and consistent? | M | S | S | M | M | M | M | S |
| Comparator: were the subjects selected from a comparable population in all respects? | M | M | S | M | M | M | W | S |
| Sample size | S | M | S | M | S | S | W | S |
| Was the follow-up timeframe sufficient? | W | M | S | S | S | S | S | S |
| Was treatment thoroughly described? | N/A | S | M | M | N/A | N/A | N/A | N/A |
| For outcomes that can vary, did the study clearly define different levels of the outcome? | M | M | S | S | S | S | S | S |
| Data Collection Method: scale, methods to collect data | M | M | M | S | M | M | M | M |
| Measurement Bias: Were the outcome measures clearly defined, valid, reliable and implemented consistently? | W | W | M | S | S | S | S | S |
| Selection Bias: Is study sample representative of target population and if <100% eligible cases were selected, were they randomized? | W | W | S | S | S | S | W | S |
| Attrition Bias: Was loss to follow-up after baseline minimized? | S | M | S | M | S | S | M | S |
| Confounders: Were key potential confounding variables measured and adjusted statistically for their impact on the relationship between treatment and outcome? | N/A | M | S | M | N/A | N/A | N/A | N/A |
| Total Points (S=3; M=2; W=1; N/A=0) | 21 | 27 | 35 | 32 | 29 | 29 | 22 | 31 |
| Overall Score (0-25 = W; 26-30 = M; >31= S) | **W** | **M** | **S** | **S** | **M** | **M** | **W** | **S** |

| CRITERIA | *Larrañaga et al., 2014* | *Richard et al.*  *2005* | *Stice et al., 2009* | *Yu, et al.,*  *2013* |
| --- | --- | --- | --- | --- |
| Research Design | M | M | M | M |
| Was the research objective clearly stated and directly related to review topic? | M | S | M | S |
| Was the study population clearly defined with inclusion/exclusion criteria stated and consistent? | S | M | S | M |
| Comparator: were the subjects selected from a comparable population in all respects? | M | M | M | M |
| Sample size | M | S | S | M |
| Was the follow-up timeframe sufficient? | S | S | S | S |
| Was treatment thoroughly described? | S | N/A | N/A | N/A |
| For outcomes that can vary, did the study clearly define different levels of the outcome? | S | S | S | S |
| Data Collection Method: scale, methods to collect data | M | M | S | M |
| Measurement Bias: Were the outcome measures clearly defined, valid, reliable and implemented consistently? | W | S | M | S |
| Selection Bias: Is study sample representative of target population and if <100% eligible cases were selected, were they randomized? | M | S | S | M |
| Attrition Bias: Was loss to follow-up after baseline minimized? | S | S | S | S |
| Confounders: Were key potential confounding variables measured and adjusted statistically for their impact on the relationship between treatment and outcome? | M | N/A | N/A | N/A |
| Total Points (S=3; M=2; W=1; N/A=0) | 30 | 29 | 29 | 27 |
| Overall Score (0-25 = W; 26-30 = M; >31= S) | **M** | **M** | **M** | **M** |

**Abbreviations.** W = weak, M = moderate, S = strong, N/A = not applicable

**Supplementary Figure S1.** Combinations of criteria used to define recovery in bulimia nervosa across the included studies
